# Mast Cell Regulation and Irritable Bowel Syndrome: Effects of Food Components with Potential Nutraceutical Use

**DOI:** 10.3390/molecules25184314

**Published:** 2020-09-20

**Authors:** José Antonio Uranga, Vicente Martínez, Raquel Abalo

**Affiliations:** 1 High Performance Research Group in Physiopathology and Pharmacology of the Digestive System NeuGut-URJC, Department of Basic Health Sciences, Faculty of Health Sciences, Universidad Rey Juan Carlos (URJC), Campus de Alcorcón, Avda. de Atenas s/n, 28022 Madrid, Spain; jose.uranga@urjc.es; 2 Department of Cell Biology, Physiology and Immunology, Neurosciences Institute, Universitat Autònoma de Barcelona, 08193 Barcelona, Spain; vicente.martinez@uab.es; 3 Center for Networked Biomedical Research on Liver and Digestive Diseases (Centro de Investigación Biomédica en Red de Enfermedades Hepáticas y Digestivas, CIBERehd), Instituto de Salud Carlos III, 28029 Madrid, Spain; 4 Associated Unit to Institute of Medicinal Chemistry (Unidad Asociada I+D+i del Instituto de Química Médica, IQM), Spanish National Research Council (Consejo Superior de Investigaciones Científicas, CSIC), 28006 Madrid, Spain

**Keywords:** cannabidiol, fatty acids, heparin, histamine, irritable bowel syndrome, mast cells, nerve growth factor, nutraceuticals, polyphenols, visceral pain

## Abstract

Mast cells are key actors in inflammatory reactions. Upon activation, they release histamine, heparin and nerve growth factor, among many other mediators that modulate immune response and neuron sensitization. One important feature of mast cells is that their population is usually increased in animal models and biopsies from patients with irritable bowel syndrome (IBS). Therefore, mast cells and mast cell mediators are regarded as key components in IBS pathophysiology. IBS is a common functional gastrointestinal disorder affecting the quality of life of up to 20% of the population worldwide. It is characterized by abdominal pain and altered bowel habits, with heterogeneous phenotypes ranging from constipation to diarrhea, with a mixed subtype and even an unclassified form. Nutrient intake is one of the triggering factors of IBS. In this respect, certain components of the daily food, such as fatty acids, amino acids or plant-derived substances like flavonoids, have been described to modulate mast cells’ activity. In this review, we will focus on the effect of these molecules, either stimulatory or inhibitory, on mast cell degranulation, looking for a nutraceutical capable of decreasing IBS symptoms.

## 1. Introduction

Amongst the many non-communicable chronic diseases, irritable bowel syndrome (IBS) is remarkable for its worldwide prevalence, variety of symptoms, diversity of etiologies, complicated diagnosis and high economic burden [1,2,3,4]. IBS has been classically described as a functional disorder of the gastrointestinal (GI) tract, in contrast with organic GI diseases (like inflammatory bowel disease, IBD), in which symptoms are explained by clear underlying pathogenic findings (namely, overt inflammation), although some overlapping has also been suggested [5]. Whereas the contribution of oxidative imbalances has been proposed more recently [6], low-grade/subtle inflammation is currently widely recognized to occur in IBS [7], leading to sensitization of local nerve fibers and, more importantly, to central sensitization [8]. Thus, IBS (like other functional GI disorders) is considered as a brain–gut axis disorder [9,10].

Although other immune cells may be involved in the pathophysiology of IBS, mast cells have been highlighted as important cell mediators of local nerve fiber sensitization [9]. Interestingly, these cells are well known for their role in the development of type 1 hypersensitization reactions, i.e., allergies, including those to different foods in particular patients [11]. However, it has been shown that specific components of food, i.e. some nutraceuticals, exert modulatory effects on these cells that may influence (increase or reduce) IBS symptoms. These nutraceuticals have been tested mostly in vitro, although some evidences have also been accumulated recently in preclinical in vivo models.

In this review, we will first describe the general features of mast cells, with particular focus on their pathophysiologic involvement in IBS. Thereafter, we will describe different nutraceuticals that have been studied for their possible modulatory effects on mast cell activity and, therefore, their potential role in triggering or inhibiting IBS symptoms. Importantly, their most likely molecular mechanisms of action will be discussed.

## 2. Mast Cells

Mast cells are immune cells with a widespread distribution. They are found in most vascularized tissues, although they are more abundant in the connective tissue of skin and mucosae, like those from respiratory, digestive and genitourinary tracts, where pathogens, allergens and other environmental agents may be encountered. In these locations, mast cells are mainly seen surrounding blood vessels, neurons or nerve fibers, muscle cells, glands and hair follicles [12,13].

Unlike other cells of hematopoietic origin derived from multipotent progenitors, mast cells do not differentiate in the bone marrow. Instead, they recapitulate the dual origin of macrophages and differentiate from yolk sac and bone marrow precursors, completing their maturation in a tissue-specific manner. This implies that there are several mast cells subtypes, depending not only on their origin but also on their final destination and expression profile [14,15,16,17]. Migration to tissues involves the expression of surface receptors and adhesion molecules, in coordination with cytoskeletal changes, to promote cell attachment to specialized regions of vascular endothelium and extravasation to particular areas of tissues. These changes vary in a tissue-specific manner according to the microenvironment of their final destination [16,17,18]. Regarding this, the gut and the respiratory tract have been the best-studied systems, with clear homing differences between them. Mast cells are abundant in the intestine, thanks to their constitutive expression of α4β7 integrin that bind to the endothelial adhesion molecule VCAM-1 (vascular cell adhesion molecule 1). On the contrary, under physiological conditions, the lung does not have a significant number of mast cell progenitors, but their numbers greatly increase during allergen-induced pulmonary inflammation, when they are actively recruited [12]. In fact, the number of mast cells in the tissues does not only depend on recruitment from blood vessels, since they can enter mitosis and proliferate in their final destinations following appropriate stimulation [19].

Although no mast cell-specific chemokine has been described so far, mast cells express several chemokine receptors which could direct their migration. Similarly, the expression of receptors like CXCR2 (chemokine (C-X-C motif) ligand 2 (Interleukin 8 receptor beta)) by endothelial cells leads to an increase of VCAM-1, which facilitates the recruitment of mast cell progenitors [18]. Another crucial factor in mast cell function is the stem cell factor (SCF) or c-kit ligand (CD117). C-kit is expressed throughout all mast cell developmental stages, from progenitors to mature cells. However, it does not seem to be involved in cell recruitment to tissues but in survival [20] and maturation of mast cells following chemotactic gradients once they reach their target organ [18]. Likewise, fibroblast membrane-bound SCF induces mast cell maturation [21]. Apart from that, other growth factors and cytokines can regulate mast cell migration and proliferation. Thus, the Th1-specific transcription factor T-bet is necessary for mast cell homing to the lung and gut. Other factors, like the Th-2-associated cytokine interleukin (IL) 4 (IL-4) and the regulatory T cells (Treg) transforming growth factor (TGF) β1 (TGF-β1) are mutually antagonistic on mast cell survival and migration; whereas IL-4 promotes survival and proliferation, TGF-β1 suppresses these processes and induces apoptosis. Other factors like IL-10, tumor necrosis factor α (TNF-α) and nerve growth factor (NGF) are also involved in mast cell physiology in an ambivalent way, since the former limits the cell migration led by TNF-α and NGF [13,18,22]. Finally, lipid mediators have also been described to play a role in mast cell maturation and homing. Bone marrow-derived mast cell (BMMC) progenitors respond chemotactically to leukotriene (LT) B4 (LTB4) but become unresponsive to LTB4 after maturation. On the contrary, prostaglandin (PG) E2 (PGE2) is an active chemotactic agent for more mature mast cells and may be involved in localization within tissues, rather than in the recruitment of progenitors from the circulation [18,23].

Once mast cells become mature, they show a distinctive feature: their large electron-dense cytoplasmic granules. The content of such granules was firstly identified as heparin and histamine [12], although mature mast cells can store and secrete a much wider variety of active products, from cytokines to proteases (Table 1). Interestingly, mast cells have been classified in humans according to their protease content: those containing predominantly tryptase (T type), which are mainly located in mucosae (i.e., colon), also known as mucosal mast cells, and connective tissue mast cells, with both tryptase and chymase as major proteases (TC type) [16,24]. It is important to note that these subtypes, and their specific proteases, may differ substantially between species, and especially between humans and rodents. This is something to consider when working with animal models [16,25]. Similarly, some plasticity can be seen between both types. In vitro studies have shown that T mast cells can alter their cytokine and protease profile after incubation with IL-4, IL-6, lipopolysaccharide (LPS) or TGF-β1 in the presence of SCF [24,26]. Also, production of heparin can be modified according to cell microenvironment in a reversible way [27,28]. Thus, the composition of granules is not homogenous, but influenced by genetic or environmental factors that modify the functional properties of mast cells [13].

In order to carry out their functions, mast cells can recognize antigens thanks to a wide range of receptors, including toll-like receptors (TLRs, indirect receptors that recognize pathogen-associated molecular patterns, PAMPs), immunoglobulin (Ig) receptors and complement, but also specific G-coupled receptors (MRGPRX2, MAS-related G protein coupled receptor-X2) for a wide range of neuropeptides and basic molecules [30,31,32]. The expression of these receptors depends on the subtype of mast cell. For example, MRGPRX2 is barely expressed in mucosal mast cells [30]. Expression of some receptors might even be inducible, as seems to be the case for TLRs (although reports on this are somehow conflicting [32,33]). After antigen binding, the response of mast cells will be also specific according to the specific receptor activated and the subtype of mast cell affected [30]. Regarding TLRs, their activation induces the nuclear translocation of nuclear factor κβ (NFκβ) in the nucleus to induce the transcription of cytokines. Particularly, TLR2 recognizes mainly PAMPs from Gram-positive bacteria, which causes the release of cytokines, such as IL-4, and histamine. LPS from Gram-negative bacteria binds to TLR4, which induces the release of pro-inflammatory cytokines (TNF-α, IL-1, IL-6) [32,34,35]. Similarly, MRGPRX2 are receptors for sensing molecules from Gram-positive bacteria triggering mast cell degranulation [35].

However, the best-studied mechanism of mast cell activation is that mediated by the IgE receptor (FcεRI, high-affinity IgE receptor) pathway. IgE antibodies are produced by mature B cells in response to CD4+ Th2 cells. They are mostly found bound to FcεRI receptors on the mast cell surface. These receptors are constitutively expressed as tetrameric receptors composed of an IgE-binding α chain, a membrane β chain and two γ chains, found as a disulfide-linked homodimer. IgE binding to FcεRI initiates phosphorylation cascades that cause degranulation, activation of transcription factors and synthesis of cytokines. Similarly, intracellular calcium concentration is increased by inositol-1,4,5-triphosphate (IP3) production, which releases calcium from the endoplasmic reticulum. Calcium activates and causes NFκB to translocate to the cell nucleus, which results again in transcription of cytokines. In addition, mast cells also express Fc receptors for IgA and IgG, although with less sensitivity [29,36,37].

The variety and specificity of the secreted products after the stimulation of mast cell receptors make these cells a main actor of the immune response. In fact, it is considered that enhancing host resistance to toxins and acute inflammation in response to pathogens might be the original function of these cells [13]. This goes beyond mast cells just being effectors of hypersensitivity reactions classically associated with allergy. They release mediators that increase vascular permeability, fluid accumulation and recruitment of immune cells, such as eosinophils, natural killer (NK) cells, neutrophils and additional mast cells [30,38], but also stimulate, through their ILs, the antigen presentation activity of dendritic cells to cytotoxic T cells. Additionally, they may also activate cytotoxic T cells directly [39]. Moreover, mast cells produce antibacterial products, such as cathelicidins and defensins, and also contribute to antiviral responses by recruiting CD8+ T cells, which produce interferon α (IFN-α) and β (IFN-β) [38]. Thus, mast cells may be considered multifunctional immune cells that, after the appropriate stimuli, mediate pro- or anti-inflammatory and/or immunosuppressive activities, both innate and adaptive, against viral, microbial and parasitic pathogens, autoimmunity and response to graft rejection, among others.

Apart from this, the variety of products that mast cells may release makes them important effectors of other non-immune functions. For instance, they stimulate keratinocytes and fibroblast during scar remodeling and reepithelization in wound healing [40]. Also, mast cells are implicated in the pathogenesis of inflammatory disorders, like atherosclerosis and aortic aneurysms, by releasing IL-6 and IFN-γ that increase the expression of matrix proteases and elastase, leading to muscle apoptosis and vascular wall remodeling [41]. During systemic hypoxia, mast cells degranulate and mediate vascular inflammatory response after reactive oxygen species (ROS) generation [38]. This mechanism of activation is also responsible for mast cell activation during the reperfusion phase after ischemia. During this process, mast cells release mediators like histamine, tryptase and chymase that increase leukocyte adhesion to endothelium and vascular permeability [42]. Their effect on endothelial cells has also been shown in certain cancers, like skin or pancreatic tumors, where they induce angiogenesis [43]. In these regards, Gounaris and collaborators have studied the effect of mast cells in colorectal cancer using an animal model defective for the adenomatous polyposis coli (APC) gene [44], a commonly mutated gene in this kind of tumors [45]. They found an increased number of mast cells at the place of polyp formation and an important remission of the lesions after mast cell depletion. However, conflicting results were obtained when APC-deficient mice were crossed with Sash mice, a mouse strain deficient for mast cells. In this case, the lack of mast cells was associated to an increase in the number and size of polyps [46]. The exact role of mast cell secretome in cancer is yet to be elucidated. However, these authors speculate that the stage of tumors could explain this result since a protective effect of the inflammatory system, by means of promoting apoptosis, is observed in early stages of tumorigenesis, while the opposite is observed at later stages, when inflammatory mediators would promote tumor progression stimulating angiogenesis [46].

Overall, mast cells display important roles in immune and non-immune functions throughout the body. Their involvement in IBS will be succinctly described next.

## 3. Mast Cells and Irritable Bowel Syndrome

Irritable bowel syndrome (IBS) is a common digestive functional disorder that seriously affects the quality of life of up to 20% of the population worldwide [1,3,47]. It is characterized by abdominal pain and altered bowel habits with heterogeneous phenotypes that range from IBS with predominant constipation (IBS-C) to IBS with predominant diarrhea (IBS-D), with a mixed subtype (IBS-M) and even an unclassified form (IBS-U) in patients who do not meet the previous criteria [2,48]. The pathogenesis of IBS is hardly understood, and the lack of tissue or molecular markers, on the one side, and that of animal models expressing all the symptoms, on the other, constitute main challenges that hamper the development of effective therapeutic approaches. However, experimental and clinical work during the last two decades has shown that altered mucosal and immune functions, enteric microbiota and nervous communication between gut and brain, play a central role in the perceptions described by patients [49].

The enteric immune system comprises a large diversity of immune cells, like mast cells, that may be sensitized and activate the inflammatory cascade in response to both extrinsic (parasites, viruses, bacteria and food) and intrinsic factors (hormones and neurotransmitters from the central nervous system, CNS). Exposure of the GI tract to an antigen also may increase fluid secretion, smooth muscle contraction and peristalsis. This highlights the intimate relationship between immune cells and enteric neurons [38].

The enteric nervous system (ENS) comprises two ganglionic plexuses. The myenteric plexus is located between the longitudinal and the circular muscle layers and is the main responsible agent controlling gut motility. The submucous plexus is located between the inner muscle layer and the mucosa and is mainly involved in interganglion communication and secretory functions. The ENS is in contact with the CNS through afferent sensory neurons and sympathetic and parasympathetic efferent neurons. Both efferent and afferent nerve fibers ramify substantially and make contact not only with enteric neurons but also with immune cells [50,51].

When an antigen permeates through the mucosa, a direct or indirect activation of mast cells’ receptors may happen, leading to degranulation and release of their mediators, as described above. The importance of mast cells in the regulation and recruitment of immune cells to the gut became highlighted when using mast cell-deficient animal models. In these animals, oral antigen sensitization did not cause immune cell infiltration in the digestive tract, unlike that observed in wild-type animals [52]. Moreover, histamine, one of the more important products secreted by mast cells, is increased in GI diseases like IBD and modulates functions of the submucous plexus, such as ion transport and neuron excitation [53,54]. Cytokines released from mast cells have pro-secretory effects in the colon [55], and the serine protease tryptase, one of the more abundant elements of mast cells secretome [56], induces the production of inflammatory mediators in IBD patients [50]. These mediators increase the excitability of enteric sensory nerves [57], which is facilitated by the close proximity between nerve endings and mast cells [58]. Indeed, it has been estimated that 90% of intestinal mucosal mast cells are in direct contact with or very close to nerves [50,58]. Similarly, mast cells can be activated by neuropeptides such as substance P (SP), resulting in the release of proteases, i.e., as a conditioned response to cold pain stress [57]. In fact, a CNS interaction with mast cells may be considered the link between stress and GI symptoms. Interestingly, in vitro observations suggest that acetylcholine promotes histamine release from mast cells, whereas degranulation and mast cell proliferation is suppressed by sympathetic activation and β2 adrenoreceptor activation [59]. Indeed, mast cells are relevant for maintaining gut homeostasis and a correct response to injury, environmental pathogens and stress [50,60]. Regarding this, stress and early adverse life events are tightly associated with IBS and even animal models of IBS have been developed under these bases [61]. In particular, rats subjected to wrap restraint stress (WRS), and pups separated from their mother, known as the maternal separation (MS) model, develop some of the typical findings of IBS, including mast cell hyperplasia close to mucosal nerve endings [62]. These effects are related to the stress-mediated release of corticotropin-releasing factor (CRF) and the subsequent activation of CRF-mediated responses. In fact, in humans, CRF acting on mast cells induces degranulation and the release of tryptase, TNF-α and histamine, resulting in visceral hypersensitivity and increased intestinal permeability, distinctive components of IBS pathophysiology. On the contrary, GI effects of stress are reduced by administration of selective CRF receptor antagonists, mast cell stabilizers and protease inhibitors [60,63,64].

Two main in vitro approaches have been assayed to study IBS: biopsies from IBS patients or cultured cells or organoids treated with extracts from human biopsies or fecal supernatants from IBS patients [61]. IBS biopsies show an increased occurrence of mast cells in the *lamina propria* compared with healthy controls [65]. Accordingly, the concentration of products from mast cells, like histamine, proteases, cytokines and PGs, is increased in mucosal biopsies and stool of IBS patients [66,67,68,69]. Interestingly, this correlates with IBS symptoms and may be the cause of the sensitization of enteric neurons and visceral afferents [66,67,68,69,70,71,72,73,74]. Similarly, mast cell mediators have also been observed to correlate with signal intensity in mesenteric afferent nerve recordings of isolated rat jejunum previously perfused with human IBS supernatants [75,76]. Sensitization has also been shown in dorsal root ganglia (DRG) neurons cultured with serine proteases or mast cell mediators released from human colonic IBS-D biopsies [76,77,78].

The importance of mast cells in intestinal nerve sensitization can be appreciated using mast cell stabilizers, like ketotifen or disodium cromoglycate (DSCG). Indeed, treatment with ketotifen significantly decreased abdominal pain, bloating, flatulence and diarrhea in IBS patients [79]. Similarly, DSCG administration resulted in a clinical improvement of symptoms in IBS-D patients after decreasing the expression of TLRs and the release of tryptase [80,81]. However, no clinical trials using these drugs are found in the ClinicalTrial.gov registry. Anti-inflammatory drugs like 5-aminosalicylic acid (5-ASA, also known as mesalamine or mesalazine) decreased the number of mast cells and their associated products of secretion, although some reports also indicate a lack of effects modulating mast cell density [82]. Despite that mesalazine has been tested in several formally registered clinical trials, its effects on colonic symptoms are not consistent [83,84]. However, the topic still raises interest and a new meta-analysis has been recently prospectively registered in PROSPERO (CRD42019147860) with the intention to provide high-quality synthesis on existing evidence for the usefulness of mesalazine on IBS [85]. Interestingly, other alternatives are being explored, like AST-10 (a carbon adsorbent capable of adsorbing low molecular substances like histamine and serotonin; ClinicalTrial.gov identifier: NCT00583128), with relatively modest results [86], or, more recently, zeolite (a volcanic mineral with absorptive properties; amongst others, the researchers will study histamine-associated readouts; ClinicalTrial.gov identifier: NCT03817645), with no results yet (currently in recruitment phase). The interference with mast cell mediators may also be an alternative for IBS patients. In this sense, the most convincing (and specific) results are those obtained with the H_1_ histamine-receptor antagonist ebastine, which decreased abdominal pain and visceral hypersensitivity in a clinical trial with 50 patients (ClinicalTrials.gov identifier: NCT01144832), whose results were published in 2016 [87]. More recently, an additional multi-center clinical trial with 200 patients was registered (ClinicalTrials.gov identifier: NCT01908465), although no further information is yet available. Although promising, the scarce number of patients in these trials preclude definitive answers and makes further replication necessary [60,88]. The message is, though, that some beneficial effects might be offered by other substances with similar mechanisms of action, including food components.

Apart from their effect on enteric nerve endings, proteases released by mast cells may also affect the integrity of the colonic mucosa. The mucosal barrier acts as a semipermeable barrier allowing the absorption of nutrients but limiting the transport of potentially harmful antigens and microorganisms. A number of studies have suggested that an increase in intestinal permeability could be a key factor of IBS progression. Indeed, the permeability of biopsies from IBS patients is increased compared to normal individuals [89,90], as also occurs with the permeability of animal mucosa samples treated with fecal supernatants from IBS patients [91]. Likewise, permeability of human cultured colonic cells was increased after incubation with supernatants of human IBS biopsies [89,90,91,92,93] or fecal supernatants from IBS patients [94].

Interestingly, the effects of proteases may be different depending on the type of IBS considered. Specifically, serine proteases levels are elevated in IBS-D patients [91]. On the contrary, cysteine proteases are predominant in the feces of the constipation variant [94]. Both degrade different adhesive proteins. Likewise, release of tryptase from mast cells increases permeability in vivo and in vitro, opening tight junctions after degrading junctional adhesion molecule (JAM), a key adhesive molecule [89,95,96]. The effect of mucosal damage may also be seen in patients suffering from post-infectious IBS (PI-IBS), a form of IBS that may occur after acute infectious gastroenteritis [97]. In this case, patients exhibit greater expression of proinflammatory products from mast cells, like IL-1β [98].

Overall, these studies clearly suggest that barrier function breakdown, with the possibility of bacterial invasion and low-grade immune system activation, plays an important role in the development of IBS symptoms, including pain hypersensitivity. This effect can be reinforced by the direct activation of mast cells in stress situations [99]. This highlights the consideration of IBS as a brain–gut axis disorder [10].

Besides the factors mentioned above, and strengthening a pivotal role of diet in IBS, compelling evidences show a link between food components, mast cells and IBS-related pathophysiology. In particular, gluten and diets rich in fermentable oligosaccharides, disaccharides, monosaccharides and polyols (FODMAPs) have received significant attention. FODMAPs are associated with the development of IBS or, at least, IBS-related symptoms, since short-chain carbohydrates pass unaltered into the colon, where they are fermented, generating gas and distention [99,100,101] and, subsequently, low FODMAPs diets have been successfully used to reduce IBS symptoms [101,102,103,104,105]. FODMAPs-induced IBS symptomatology might involve the recruitment and activation of mast cells [99]. Indeed, recent pre-clinical data show that the effects of FODMAPs diets in different animal models of IBS might be related to an increase in colonic mast cells [106,107]. Moreover, changes in histamine levels, one of the key mediators released during mast cell activation, are reduced in IBS patients under a low FODMAPs diet, thus indicating a potential modulation of mast cell activity [104].

A role for gluten in IBS pathophysiology is supported by the fact that a gluten-free diet ameliorates IBS symptomatology, while IBS symptoms are induced following the ingestion of gluten in patients with IBS [100,108]. However, there are questions regarding which components of wheat are implicated in these responses and the underlying mechanisms. In any case, mast cells have a relevant role in the pathophysiology of celiac disease [109], thus indicating that they might also be involved in the generation of IBS symptomatology.

Other dietary interventions have focused on different carbohydrates or fiber composition (like the paleo diet, the specific carbohydrate diet and the diet for sucrose-isomaltase deficiency), on proteins (such as the reduced resistant protein diet), or on bioactive molecules (such as the low amine/histamine diet, the low capsaicin diet and the low food chemical diet). Although further investigation is needed for the use of these diets in the clinical practice, it seems clear that there is a large array of potential harmful molecules for patients with IBS [110].

Altogether, these observations further support the view that mast cells should be regarded as a target in the treatment of IBS, subjected to potential diet/nutraceutical interventions.

## 4. Nutraceuticals Affecting Mast Cell Activity

Mast cells’ activity can be modulated by different stimuli, with stimulation via the FcεRI being the best-established activation signal. Since aggregation of IgE and subsequent FcεRI activation on mast cells initiates type I allergic reactions, nutrient-associated modulation of mast cells has been directed mainly towards allergic reactions [111,112]. However, mast cell activation and degranulation, as well as synthesis of mediators, can also be modulated by several IgE-FcεRI-independent mechanisms [113]. In any case, although functional and inflammatory GI disorders are not allergic processes, immune-related mechanisms linked to dietary antigens might have relevance in their pathophysiology [114,115,116,117,118]. Moreover, connection between IgE-mediated responses and IBS may be not only local, but also systemic. Indeed, IBS prevalence is higher in patients suffering from atopic IgE-dependent diseases than in healthy populations, and atopic diseases could predispose to developing IBS [118].

Numerous food components have been shown to manifest immunomodulatory capacities as it relates to mast cell functioning, acting as modulators of mast cell activation/degranulation or/and modulating the synthesis of mediators (see Table 2, Table 3, Table 4, Table 5, Table 6, Table 7 and Table 8). The bioactivity of these nutrients could be used by applying them as nutraceuticals in the context of diverse mast cell-associated diseases through the downregulation of mast cell activation. Given the key role played by mast cells in GI functional, particularly IBS [119,120], and inflammatory disorders [121,122], a potential application of these nutraceuticals is envisaged in the management of these conditions. It is important to note that most of the data available so far derives from in vitro observations in relevant systems (different human- and rodent-derived mast cell lines and isolated mast cells), with only a few in vivo studies in relevant animal models or clinical trials. Indeed, the clinical trials performed so far are very limited and a direct relationship between mast cells and the possible positive effects observed at a clinical level cannot be established. Thus, such a link can only be hypothesized, so far, taking into account preclinical, in vitro and in vivo, observations.

### 4.1. Lipids

Lipid-rich enteral feeding significantly decreased circulatory levels of mouse mast cell protease, compared with isocaloric low-lipid nutrition or fasting, thus indicating a potential role for diet-derived lipids as immunomodulatory agents with effects on intestinal mast cells’ activity [123]. Although the mechanisms of action are not fully elucidated, direct effects of immunomodulatory lipids on mast cells’ degranulation, changes in local lipid composition and changes in lipid transport affecting mast cells’ reactivity are possible mechanisms by which the function of mast cells might be modulated by diet-derived lipidic compounds [124]. These actions open the use of specific lipids as nutraceuticals with the aim of reducing mast cell activity and therefore the undesired neuro-immune-endocrine responses associated.

#### 4.1.1. Fatty Acids

Evidences indicate that different fatty acids are able to modulate the synthesis and release of mast cell mediators [125]. Effects observed were fatty-acid-specific and included both facilitation and inhibition of release, depending upon the mediators considered (Table 2). Main evidences derive from in vitro studies based on the incubation of human mast cell lines (mainly LAD-2 and HMC-1) or BMMCs with different fatty acids, assessing the release of different mediators. In this respect, the n-6 long-chain polyunsaturated fatty acid (PUFA) arachidonic acid (AA) or the n-3 long-chain PUFAs eicosapentaenoic acid (EPA) or docosahexaenoic acid (DHA) affected mast cell activation, although they did not affect IgE-mediated mast cell degranulation [126]. Similarly, α-linolenic acid (ALA) and its metabolites, including EPA and DHA, decreased the in vitro production of ILs [127] and PGD2 [128]. In a different study, EPA and DHA reduced TNF-α release from HMC-1 cells but did not affect degranulation [129]. Overall, n-3 long-chain PUFAs were associated with anti-inflammatory/antiallergic effects, while n-6 long-chain PUFAs seem to be related with proinflammatory/proallergic responses.

Similar modulatory activity was observed in a canine mastocytoma cell line (C2). Specifically, γ-linolenic acid (GLA) (n-6) increased tryptase activity and decreased histamine release in stimulated C2 cells and DHA (n-3) reduced PGE2 production. On the other hand, ALA (n-3) caused a reduction of tryptase activity, PGE2 production as well as histamine release, while linoleic acid or AA (n-6) increased them [130,131,132]. No effects were observed on chymase activity. Thus, ALA (n-3) exhibited specific anti-inflammatory effects, at least as it relates to cultured canine mastocytoma cells.

In an in vivo approach, a diet rich in n-6 linoleic acid, saturated fatty acids (safflower oil), but not monounsaturated fatty acids (coconut oil) or n-3 PUFAs (fish oil), reduced circulatory release of chymase II, as a marker for degranulation of mucosal mast cells, in an intestinal mast cell-IgE-mediated inflammatory reaction model in rats [133]. These effects were associated with an enrichment of linoleic acid in the mast cell membrane, which altered the membrane structure and resulted in a reduced number and/or affinity of IgE receptors. However, a direct inhibitory effect of linoleic acid or its metabolites on IgE-mediated degranulation might also be possible [133]. These observations were further confirmed in a murine atopic model in which oral administration of fish oil, containing high levels of omega-3 fatty acids, significantly reduced the severity of dermatitis and the thickening of epidermis/dermis [127]. However, in a model of stress-induced visceral hypersensitivity in maternally-separated rats, a model associated with mast cell hyperactivity and, as previously mentioned, regarded as relevant for the study of IBS pathophysiology, a diet enriched in n-3 PUFAs (tuna oil) did not affect hypersensitivity nor mast cell degranulation [129].

Short-chain fatty acids (namely acetate, propionate and butyrate) have been suggested to modulate inflammatory responses within the gut, including the inhibition of the release of mast cell-derived proinflammatory mediators [134,135]. Following these observations, in an in vivo study, sodium butyrate supplementation improved intestinal health in pigs, an effect associated with a reduction in the percentage of degranulated mast cells and the content of its inflammatory mediators (histamine, tryptase, TNF-α and IL-6) in the mucosa of the jejunum. Moreover, a reduction in mast cell expression of tryptase, TNF-α and IL-6 was also observed [136].

#### 4.1.2. Cannabinoids, Cannabinoid-Related Compounds and Other Lipidic Molecules

Cannabidiol is a non-psychoactive cannabinoid with positive effects on intestinal health that has been suggested as a potential nutraceutical because of its effects on the endocannabinoid system (see Reference [137] for a recent review on the topic). In a murine model of LPS-induced intestinal inflammation, cannabidiol prevented the associated upregulation of mast cell chymase and matrix metalloproteinase (MMP) 9 (MMP9), thus suggesting a potential anti-inflammatory effect mediated, at least partially, through the modulation of mast cell activity [138].

Palmithoylethanolamide, the saturated fatty acid amide of palmitic acid, is a dietary component commonly found in egg yolk and peanuts, structurally related to the endocannabinoid anandamide. Palmithoylethanolamide has been considered as an endogenous modulator of mast cell activation (see Reference [139] for review). Palmithoylethanolamide modulated the activity of the endocannabinoid system in mast cells, thus potentiating its beneficial effects on inflammation. Moreover, palmithoylethanolamide has been shown to prevent IgE-induced degranulation in isolated canine skin mast cells (histamine, PGD2 and TNF-α release) [140]. Similar results were also observed in human mast cells (HMC-1), where palmithoylethanolamide prevented NGF release [141]. These in vitro evidences agree with in vivo observations showing that palmithoylethanolamide was able to control mast cell-derived inflammation in immunogenic and non-immunogenic animal models of disease [142,143,144,145]. Overall, positive effects of palmithoylethanolamide were associated with a reduction of the production and release (degranulation) of several mediators, such as TNF-α and neurotrophic factors, like NGF, and proteases (tryptase and chymase) [146,147]. From these evidences, several studies have assessed the utility of palmithoylethanolamide in inflammatory and pain syndromes in both animals and humans (see Reference [139] for review). In some cases, the clinical improvement of symptoms was clearly correlated with the control of mast cell activation [148]. A recent clinical trial assessed the analgesic properties of dietary supplementation with palmitoylethanolamide and polydatin in IBS, reporting an improvement of abdominal pain severity (ClinicalTrials.gov number, NCT01370720) [149]. However, no changes in mast cells numbers or in the mast cell activation profile were observed [149]. Therefore, the link between the positive clinical effects and the potential modulation of mast cells is still a question and further studies are required to elucidate the mechanism of action of palmitoylethanolamide/polydatin in IBS.

Sphingolipids, and particularly ceramide and sphingosine, have been shown to negatively regulate mast cell signals and function [150]. In particular, they inhibited cytokine production from mast cells in culture [151] and induced apoptotic cell death in mouse BMMCs [150]. On the other hand, sphingosine-1-phosphate exhibits positive regulatory actions, enhancing mast cell function, including LT synthesis, TNF-α production, chemokine production and β-hexosaminidase release [150,152].

Table 3 offers a summary of the compounds mentioned in this section and their effects on mast cells’ activity.

#### 4.1.3. Fat-Soluble Vitamins

Table 4 summarizes the immunomodulatory effects exerted by vitamins D and E on mast cell activity.

Vitamin D is necessary to maintain the stability of mast cells, which activate automatically in a vitamin D-deficient environment, in the absence of specific activators. Exposure to vitamin D3 (calcitriol) resulted in an increased expression of vitamin D receptors and repressed the expression of TNF-α in different mast cell lines [153]. In accordance with these observations, sensitized mice receiving a vitamin D-supplemented diet showed reduced levels of serum histamine and TNF-α when challenged with the sensitizing antigen, thus indicating a hampered mast cell activation and a protective role for vitamin D [153].

Inefficiently absorbed vitamin E analogues could be considered as preventive nutraceuticals against intestinal inflammatory and allergic events and colon cancer. Vitamin E, and in general tocopherol analogues, have been shown to inhibit proliferation and survival of mast cells, likely affecting components of the c-kit/PI3K/PKB signaling cascade [154]. Several in vitro studies using different mast cell lines have also demonstrated that vitamin E modulates degranulation of mast cells, leading to a reduction in proinflammatory mediators, including histamine and PGD2 release, and a decrease in chymase activity, whereas tryptase activity was not affected [155,156]. Overall, these effects might be associated with the anti-free-radical and antioxidative stress actions of tocopherols [154].

### 4.2. Amino Acids

Experimental data have shown that human intestinal mast cells respond to the stimulation with specific amino acids, namely arginine and glutamine (Table 5). Arginine and glutamine are considered conditionally essential amino acids, mainly in stages of metabolic stress in the gut. In particular, it has been shown that pharmacological doses of arginine in combination with glutamine exert protective effects, for example, in Crohn’s disease (CD), by reducing the production of proinflammatory cytokines such as TNF-α, IL-6 and IL-8 [157]. Studies in mature human mast cells isolated from normal surgery tissue specimens showed that a combination of both amino acids at pharmacological doses reduced LTC4 secretion and the expression of the chemokines CCL2, CCL4, IL-8 and TNF-α [158]. These observations suggest that the beneficial effects previously observed might be associated, at least partially, to a direct effect modulating intestinal mast cells’ activity.

Recent data showed that dietary asparagine supplementation ameliorated LPS-induced intestinal dysfunction in pigs in in vivo conditions [159]. Although a direct effect on mast cells was not assessed, a reversion in the increase in intestinal mast cells triggered by LPS was observed, thus indicating a potential effect preventing mast cell-mediated actions within the gut. Further studies are needed to determine if asparagine, in addition to mast cell density, is able to also modulate mast cell activity/degranulation.

In a murine model of allergy to cow’s milk, oral administration of glycine modulated mast cell-dependent allergic responses (as denoted by a reduction in plasma levels of mouse mast cell protease-1) and normalized the intestinal density of mast cells [160]. These effects on allergic mechanisms suggest that glycine might have a potential nutraceutical application modulating mast cell activity within the gut.

### 4.3. Carotenoids

Carotenoids are a heterogeneous group of natural pigments with diverse biological functions, including anti-oxidative and anti-inflammatory activities, which might affect mast cell function and, therefore, have an impact on GI physiology and pathophysiology. Numerous carotenoid compounds have been shown to modulate the activity of mast cell-related cell lines in in vitro conditions (Table 6). These include compounds such as fucoxanthin, astaxanthin, zeaxanthin or α- and β-carotene. Overall, these compounds inhibited antigen-induced degranulation [161] and histamine release [162]. These in vitro findings correlate with in vivo observations showing negative modulatory effects on mast cell function. For instance, α- and β-carotene treatment inhibited allergic responses, including the rise in serum histamine associated with mast cell activation [163]. Similarly, astaxanthin reduced signs of inflammation and the levels of TNF-α and IFN-γ in a dinitrofluorobenzene (DNFB)-induced contact dermatitis mouse model [162].

Retinol has been suggested as a negative regulator for the differentiation of human mast cells [164,165,166]. However, no effect [165] or enhanced degranulation has been observed upon incubation of mature mast cells with vitamin A [167]. Therefore, additional studies are necessary to determine the potential nutraceutical effects of retinol modulating the activity of intestinal mast cells and/or the maturation/differentiation process of mast cells arriving to the gut.

### 4.4. Polyphenolic Compounds

#### 4.4.1. Flavonoids

Flavonoids (or bioflavonoids) are a family of naturally occurring polyphenolic plant and fungus substances with antioxidative, anti-cancer and anti-inflammatory properties. They are naturally found in fruits, vegetables, herbs, nuts, spices and red wine, with low toxicity compared to other active plant compounds. One group of flavonoids, the flavonols (3-hydroxyflavone), has been shown to have beneficial effects on mast cells (Table 7).

Several flavonoids (such as flavone, luteolin, fisetin, quercetin, rutin, kaempferol, myricetin, caffeic acid, nobiletin or morin) decreased the expression and/or inhibited the release of pro-inflammatory cytokines (TNF-α, IL-1, IL-6, and CXCL8, CCL2, CCL3 and CCL4), cysteinyl LTs and PGD2, as well as of tryptase, β-hexosaminidase and histamine in human and rodent mast cells in in vitro conditions [168,169,170,171,172,173,174,175,176,177,178]. These modulatory effects were both IgE-dependent and independent and exhibited compound-dependent selectivity and potency; for instance, morin was significantly less potent than other flavonoids modulating the activity of rat basophilic leukemia (RBL) cells [170].

In the murine IL-10 knockout model of colitis, treatment with the citrus flavonoid nobiletin resulted in a reduction of clinical colitis and a reduction of mast cell number and degranulation, which correlated positively with disease activity indexes [179].

Fermented soy germ-derived phytoestrogens, containing daidzein, glycitein and genistein present in aglycone forms, represent gut absorbable isoflavone forms structurally related to 17β-estradiol, but with higher affinity for estrogen receptors [180]. In a model of stress-induced IBS-like symptoms in female rats, supplementation with these soy germ fermented ingredients prevented the development of visceral hypersensitivity and intestinal barrier alterations characteristic of IBS. A reduction in colonic mast cell density and fecal proteolytic activity was also observed, thus suggesting that the functional changes observed might be associated with a modulation of mast cell activity [181].

Catechins (a family of flavonols) also play a critical role influencing mast cell activation, especially their derivative epigallocatechin-3-gallate (EGCG), a major green tea polyphenol [182,183,184]. Both, IgE-dependent and independent effects have been implicated in catechins effects, with some contradictory observations. EGCG inhibited histamine release from RBL-2H3 cells [185,186] and reduced degranulation (β-hexosaminidase release) and LTC4 secretion from RBL-2H3 cells as well as BMMCs upon IgE-dependent stimulation [182]. However, EGCG induced cytokine production (IL-13 and TNF-α) in mast cells (RBL-2H3 cells and BMMCs) via Ca^2+^ influx and ROS generation [183].

Flavanones, particularly hesperetin and naringenin, are also important bioactive components of citric fruits. Hesperetin and naringenin suppressed degranulation in RBL-2H3 cells, leading to the suppression of cytokines [187].

#### 4.4.2. Other Polyphenolic Compounds

Not yet characterized polyphenolic compounds [188] are likely to mediate the beneficial effects observed in vivo for royal jelly in a murine model of cow milk allergy [189]. In this model, oral administration of royal jelly reduced histamine levels and prevented the associated intestinal damage, at least partially due to the activation of mast cells [189].

### 4.5. Spices

Two spices or their derived compounds have shown interesting roles related with immunomodulation of mast cell activity (Table 8): curcumin and cinnamon.

#### 4.5.1. Curcumin

Curcumin is a diarylheptanoid phytochemical, belonging to the group of curcuminoids, which are natural bioactive phenols derived from the rhizome (turmeric) of *Curcuma longa* plants [190]. In vitro (BMMCs and RBL-2H3 cells) and in vivo studies (passive cutaneous anaphylaxis in mice) have shown that curcumin inhibited antigen-mediated activation of mast cells by suppressing degranulation and secretion of TNF-α and IL-4 [191]. Similar positive effects were also observed in a model of food-induced, IgE-mediated intestinal inflammatory reaction in rats, in which curcumin supplementation reduced mast cell activity [133]. Several clinical studies support a role for curcumin in inflammatory and functional GI diseases. In a randomized, double-blind placebo-controlled study in patients with ulcerative colitis, curcumin (plus sulfasalazine or mesalamine) improved the clinical activity index and the endoscopic score and prevented acute ulcerative colitis flares [192]. Similarly, in a randomized controlled clinical trial, curcumin (plus mesalazine) induced remission in patients with mild-to-moderate ulcerative colitis (ClinicalTrials.gov identifier: NCT01320436) [193]. As it relates to IBS, in a small group of patients [194], curcumin decreased abdominal pain intensity and improved quality of life. A combination of curcumin with fennel essential oil (with anethole as the active component) has also shown an improvement of symptoms and quality of life in IBS patients [195]. However, there is no evidence linking these positive clinical effects with the modulation of mast cell activity. The interest in curcumin is also highlighted by the fact that, according to the ClinicalTrial.gov registry, two additional clinical trials on IBS have been completed (ClinicalTrials.gov identifiers: NCT00779493 and NCT01418066), although no data have been released.

#### 4.5.2. Cinnamon Extract—Cinnamaldehyde

Cinnamon extract treatment on IgE-stimulated RBL-2H3 cells as well as on human intestinal mast cells caused a downregulation of degranulation and *de novo* synthesis of proinflammatory mediators (CXCL8, CCL2, CCL3, CCL4 and TNF), β-hexosaminidase and cysteinyl LTs, as well as tryptase expression [196]. For RBL-2H3 cells, the IgE-independent activation was also detected, although to a lower extent [196]. Moreover, oral cinnamon extract treatment caused a downregulation of tryptase and carboxypeptidase A3 (MC-CPA) expression [196] and reduced expression of mast cell proteases (MC-CPA, MCP-1 and MCP-4) and pro-inflammatory mediators (CXCL8, CCL2, CCL3 and CCL4) during colitis in IL-10 knockout mice [197]. A subsequent study identified cinnamaldehyde as the main mediator of cinnamon extract in mast cell inhibition [198].

## 5. Conclusions

**Figure 1:**
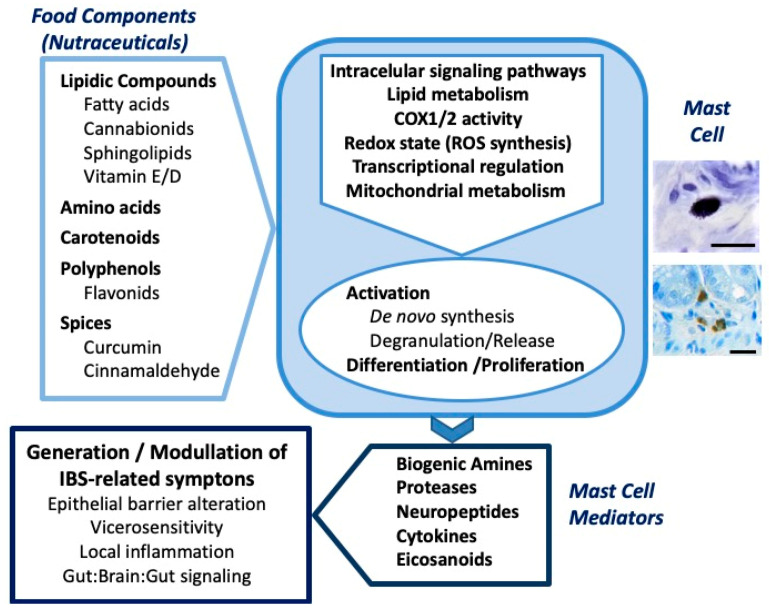
Schematic representation of the modulatory activity of food components on mast cells and their relationship with the generation and modulation of irritable bowel syndrome (IBS)-related symptoms. The figure integrates the main bioactive food components with potential nutraceutical use (as discussed in the text), the main mechanisms of action proposed on mast cells and some of the key symptoms related to IBS that can be modulated through the action of mast cell-derived mediators. See text for details. The photomicrographs (right) show mast cells in the rat intestine, identified with toluidine blue staining (upper photomicrograph) and rat mast cell protease 2 immunohistochemistry (lower photomicrograph). Scale bar: 20 μm.

Mast cells play a prominent role in the pathophysiology of functional GI disorders, particularly IBS. Therefore, they have been regarded for a long time as a pharmacological target for the control of IBS. However, the paucity in the development of specific drugs targeting mast cells, or affecting IBS pathophysiology in general, has increased the interest in the search of alternative treatments. In this context, nutrient-derived bioactive compounds, administered as nutraceuticals, might represent a feasible alternative to the traditional pharmacological approach. Consistent in vivo and in vitro evidences indicate that numerous nutrient-derived bioactive compounds (including a variety of lipidic compounds, amino acids or numerous polyphenolic compounds) have the ability to modulate mast cell activity in a specific manner, reducing the release (mast cell degranulation) and the *de novo* synthesis of mast cells’ mediators considered to mediate, at least in part, the neuro-immune-endocrine alterations present in IBS (Figure 1). Nevertheless, the clinical evidences are still scarce and additional studies are necessary to clearly show validity of this approach and the efficacy of nutraceuticals for the treatment of IBS, and, in particular, modulating mast cell activity. Moreover, it is expected that during the coming years, additional studies, both in vitro, in cellular systems and organoid human cell cultures, and in vivo, in disease-relevant animal models, will contribute to the identification of new food-derived bioactive compounds with potential nutraceutical applications, including the negative modulation of mast cells.

## Figures and Tables

**Table 1:** Molecules that may be secreted by mast cells ^a^.

Category	Specific Molecules
Biogenic amines	Histamine, 5-HT, Dopamine, Polyamines
Lysosomal Enzymes	β-hexosaminidase, β-glucuronidase, β-d-galactosidase, Arylsulphatase A, Cathepsins
Proteases	Chymase, Tryptase, Carboxypeptidase A, Granzyme B, MMPs, Renin
Other Enzymes	Kinogenases, Heparanase, Angiogenin, Caspase-3, COX 1 and 2
Proteoglycans/Glycosaminoglycans	Serglycin, Heparin
Cytokines	TNF, IL-1, IL-2, IL-3, IL-4, IL-5, IL-6, IL-8, IL-9, IL-10, IL-11, IL-12, IL-13IL-15, IL-16 IL-17, IL-18, IL-25, IL-33, IFN, MIP-1α and 2β
Chemokines	RANTES (CCL5), eotaxin (CCL11), MCP-1 (CCL2), MCP-3 (CCL7), MCP-4
Growth Factors	TGF-β, VEGF, NGF, SCF, GM-CSF, FGF, NGF, PDGF, LIF
Peptides	CRF, Endorphin, ET-1, Cathelicidin (LL37), Defensins, SP, VIP
Phospolipid Metabolites	PGD2, PGE2, LTB4, LTC4, PAF
Reactive Oxygen Species	NO
Others	MBP, Complement Factors C3 and C5

^a^ Adapted from References [12,29]. See abbreviations at the end of the article.

**Table 2:** Immunomodulatory effects of fatty acids on mast cell activity.

Compound	System	Effect ^a^	Mechanism of Action	Reference
**In Vitro Studies**				
AA (20:4n-6)	LAD2 HMC-1	↑ PGD2 ↑ TNF-α	ROS generation and MAPK signaling	[126]
AA (20:4n-6)	C2	↑ Tryptase activity ↑ PGE2 production ↑ Histamine release	Changes in cellular redox state and lipid peroxidation (suggested)	[132]
ALA (18:3n-3)	MC/9, BMMCs	↓ IL-4, IL-5 and IL-13 production	Modulation of nuclear expression of GATA-1 and GATA-2	[127]
ALA (18:3n-3)	C2	↓ Tryptase activity ↓ PGE2 production ↓ Histamine release		[130,131]
DHA (226n-3)	LAD2 HMC-1	↓ Il-4 ↓ IL-13 ↓ ROS generation	MAPK signaling	[126]
DHA (22:6n-3)	HMC-1	↓ TNF-α release	PPARγ-dependent activation	[129]
EPA (20:5n-3)	LAD2 HMC-1	↓ Il-4 ↓ IL-13 ↓ ROS generation	MAPK signaling	[126]
EPA (20:5n-3)	Mast cells cultured from human umbilical cord mononuclear cells	↓ PGD2 generation	Inhibition of COX-1 and COX-2 activities	[128]
EPA (20:5n-3)	MC/9, BMMCs	↓ IL-4, Il-5 and IL-13 production	Modulation of nuclear expression of GATA-1 and GATA-2	[127]
EPA (20:5n-3)	HMC-1	↓ TNF-α release	PPARγ-dependent activation	[129]
EPA (20:5n-3)	MC/9, BMMCs	↓ IL-4, Il-5 and IL-13 production	Modulation of nuclear expression of GATA-1 and GATA-2	[127]
EPA (20:5n-3)	C2	↑ PGE2 production ↑ Histamine release	Changes in cellular redox state and lipid peroxidation (suggested)	[132]
GLA (18:3n-6)	C2	↑ Tryptase activity ↑ Histamine release		[130,131]
**In Vivo Studies**				
Diet rich in n-6 linoleic acid, saturated fatty acids (safflower oil)	Intestinal mast cell-IgE-mediated inflammatory reaction model in rats	↓ Rat chymase II		[133]
Fish oil containing high level of omega-3 fatty acids	NC/Nga murine atopic model.	↓ Severity of dermatitis ↓ Thickening of epidermis/dermis		[127]
Sodium butyrate (SCFA)	Pig	↓ Histamine content ↓ Tryptase content/expression ↓ TNF-α content/expression ↓ IL-6 content/expression	JNK signaling pathways	[136]

^a^: ↑: Facilitation; ↓: Inhibition. See abbreviations at the end of the article.

**Table 3:** Immunomodulatory effects of cannabinoids, cannabinoid-related compounds and other lipidic molecules on mast cell activity.

Compound	System	Effect ^a^	Mechanism of Action	Reference
**Cannabinoids and Cannabinoid-Related Compounds**				
Cannabidiol	LPS-induced intestinal inflammation in mice	↓ Chymase up-regulation ↓ MMP9 up-regulation	Involvement of astroglial signaling neurotrophin S100B and PPARγ-dependent mechanisms	[138]
Palmithoylethanolamide	Canine skin mast cells	↓ Histamine release ↓ PGD2 release ↓ TNF-α release		[140]
Palmithoylethanolamide	HMC-1	↓ NGF release	GPR55-mediated	[141]
Palmithoylethanolamide	Neuropathic pain (chronic constriction injury of sciatic nerve in mice)	↓ TNF-α release ↓ NGF release		[146]
Palmithoylethanolamide	Spinal cord injury (mice)	↓ Proteases (tryptase and chymase) release		[147]
Palmithoylethanolamide/Polydatin	Clinical trial in IBS patients (NCT01370720)	Without changes in mast cell counts		[149]
**Other Lipidic Molecules**				
Ceramide/sphingosine	Mouse BMMCs	↓ IL-5, IL-10 and IL-13 production	Inhibition of PI3K-Akt pathway	[151]
Sphingosine-1-phosphate	Mouse BMMCs RBL-2H3 cells (rat)	↑ LT synthesis ↑ TNF-production ↑ Chemokines production ↑ β-hexosaminidase release	FcεRI-mediated activation of SphK-S1P1/S1P2 pathway	[150,152]

^a^: ↑: Facilitation; ↓: Inhibition. See abbreviations at the end of the article.

**Table 4:** Immunomodulatory effects of fat-soluble vitamins on mast cell activity.

Compound	System	Effect ^a^	Mechanism of Action	Reference
Vitamin D3 (calcitriol)	HMC-1 cells (human) RBL-2H3 cells (rat) p815 cells (mouse) Mouse BMMCs	↓ TNF-α expression ↓ TNF-α production ↓ Histamine release	Inhibition of FcεRI and MyD88, associated to decreased Syk phosphorylation and MAPK and NFκB levels. VDR binding to the TNF-α promoter leading to decreased acetylation of histone H3/H4, RNA polymerase II and OCT1 (a transcription factor of TNF-α) at the promoter locus, repressing TNF-α expression	[153]
Vitamin D3 (calcitriol)	Ovalbumin –sensitized mice with vitamin D-supplemented diet	↓ Serum TNF-α ↓ Serum histamine		[153]
Vitamin E (tocopherols)	C2 (canine)	↓ Histamine release ↓ PGD2 release ↓ Chymase activity		[155]
Vitamin E (tocopherols)	Rat peritoneal mast cells	↓ Histamine release	Changes in lipid peroxidation through the lipoxygenase pathway	[156]

^a^: ↑: Facilitation; ↓: Inhibition. See abbreviations at the end of the article.

**Table 5:** Immunomodulatory effects of amino acids on mast cell activity.

Compound	System	Effect ^a^	Mechanism of Action	Reference
Arginine + Glutamine	Human intestinal mast cells	↓ LT C4 secretion ↓ CCL2 expression ↓ CCL4 expression ↓ IL-8 expression	Decreased activation levels of signaling molecules of the MAPK family (extracellular signal-regulated kinase, JNK and p38) and the Akt	[158]
Glycine	Murine model of allergy to cow’s milk	↓ Plasma levels of mouse mast cell protease-1		[160]

^a^: ↑: Facilitation; ↓: Inhibition. See abbreviations at the end of the article.

**Table 6:** Immunomodulatory effects of carotenoids on mast cell activity.

Compound	System	Effect ^a^	Mechanism of Action	Reference
Carotenoids (fucoxanthin, astaxanthin, zeaxanthin and β-carotene)	Rat RBL-2H3 cells Mouse BMMCs	↓ β-hexosaminidase release	Inhibition of FcεRI-mediated intracellular signaling: phosphorylation of Lyn kinase and Fyn kinase	[161]
α- and β-carotene	Ovalbumin–sensitized mice	↓ Histamine release		[163]
Astaxanthin	DNFB-induced contact dermatitis in mice	↓TNF-α levels ↓ IFN-γ levels		[162]
Astaxanthin	Rat RBL-2H3 cells	↓ Histamine release ↓ β-hexosaminidase		[162]

^a^: ↑: Facilitation; ↓: Inhibition. See abbreviations at the end of the article.

**Table 7:** Immunomodulatory effects of polyphenolic compounds on mast cell activity.

Compound	System	Effect ^a^	Mechanism of Action	Reference
Quercitin	RBL-2H3 cells	↑ Rat mast cell protease II synthesis ↑ Accumulation of secretory granules ↓ Histamine release ↓ β-hexosaminidase release		[168,170]
Flavone	RBL-2H3 cells	↑ Accumulation of secretory granules ↓ β-hexosaminidase release		[168]
Kaempferol	RBL-2H3 cells	↓ β-hexosaminidase release		[168]
Myricetin	RBL-2H3 cells	↓ β-hexosaminidase release		[168]
Luteolin, baicalein, quercetin	BMMCs Rat peritoneal mast cells	↓ Histamine release ↓ Il-6 production ↓ TNF-α production		[171]
Luteolin, baicalein, quercetin	Human cultured mast cells	↓ Histamine release ↓ LTs release ↓ PGD2 release	Inhibition of Ca^2+^ influx and PKC, ERKs and JNK signaling pathways	[172]
Kaempferol, myrecitin, quercetin, rutin, fisetin	RBL-2H3 cells HMC-1 cells	↓ Histamine release ↓ TNF-α expression and release ↓ IL-1β expression and release ↓ IL-6 expression and release ↓ Il-8 expression and release	Suppression of NFκB activation (fisetin, myricetin and rutin)	[174]
Quercetin, kaempferol, 14yricetin, morin	Human umbilical cord BMMCs	↓ Histamine release ↓ TNF-α release ↓ IL-6 release ↓ IL-8 release	Suppression of intracellular Ca^2+^, inhibition of PKC θ phosphorylation	[175]
Nobiletin, tangeretin	Human intestinal mast cells	↓ CXCL8 expression ↓ CCL3 expression ↓ CCL4 expression ↓ IL-1β expression (tangeretin) ↓ TNF-α expression ↓ β–hexosaminidase release (nobiletin) ↓ cysteinyl LTC4 (nobiletin)	Reduced phosphorylation of ERK1/2	[177]
Nobiletin	Murine IL-10 knockout model of colitis	↓ Mast cell density (colon) ↓ Mast cell degranulation (colon)		[177]
Daidzein, glycitein and genistein	Restraint stress-induced IBS-like alterations in rats	↓ Colonic mast cell density	Estrogen receptor-mediated	[181]
Green tea polyphenols	RBL-2H3 cells	↓ Histamine release	Metabolic events associated to the elevation of intracellular Ca^2+^, inhibition of tyrosine phosphorylation of cellular proteins including pp125(FAK)	[185,186]
Green tea polyphenols	RBL-2H3 cells BMMCs	↓ β-hexosaminidase release ↓ LTC4 secretion	Changes in ROS production and mitochondrial membrane potential	[182]
Green tea polyphenols (EGCG)	RBL-2H3 cells BMMCs	↑ IL-13 production ↑ TNF-α production	SOC-dependent Ca^2+^ influx and ROS generation	[183]

^a^: ↑: Facilitation; ↓: Inhibition. See abbreviations at the end of the article.

**Table 8:** Immunomodulatory effects of spices on mast cell activity.

Compound	System	Effect ^a^	Mechanism of Action	Reference
Curcumin	Intestinal mast cell-IgE-mediated inflammatory reaction model in rats	↓ Rat chymase II		[133]
Curcumin	RBL-2H3 cells BMMCs	↓ TNF-α expression and release ↓ IL-4 expression and release ↓ β –hexosaminidase release	Inhibition of Syk activity, inhibition of phosphorylation of Akt and MAPKs p38, p44/42 and JNK	[191]
Curcumin	Passive cutaneous anaphylaxis model in mice	↓ Mast cell-dependent passive cutaneous anaphylaxis responses (Evans blue extravasation)		[191]
Cinnamon extract/Cinnamaldehyde	Human intestinal mast cells RBL-2H3 cells	↓ Tryptase expression ↓ β–hexosaminidase release ↓ cysLt release ↓ CXCL8 release ↓ CXCL8 expression ↓ CCL2 expression ↓ CCL3 expression ↓ CCL4 expression ↓ TNF-α expression	Inhibition of Akt and the MAPKs ERK, JNK, and p38; inhibition of PLCγ1 phosphorilation	[196,198]
Cinnamon extract/Cinnamaldehyde	Mouse duodenal tissue	↓ MCP6 and MC-CPA expression		[196]
Cinnamon extract/Cinnamaldehyde	Murine IL-10 knockout model of colitis	↓ Proteases expression (MC-CPA, MCP-1 and MCP-4) ↓ Expression of pro-inflammatory mediators (CXCL8, CCL2, CCL3 and CCL4, IL-1β, TNF, INFγ)	Inhibition of NFκB signaling	[197]

^a^: ↑: Facilitation; ↓: Inhibition. See abbreviations at the end of the article.

## Abbreviations

5-ASA	5-aminosalicylic acid
5-HT	serotonin
AA	arachidonic acid
ALA	α-linolenic acid
Akt	protein kinase B
APC	adenomatous polyposis coli
BMMCs	bone marrow-derived mast cells
C2	canine mastocytoma cell line
CCL	C-C motif chemokine ligand
CD	Crohn’s disease
CNS	central nervous system
COX	cyclooxygenase
CRF	corticotropin releasing factor
CXCL	Chemokine (C-X-C motif) ligand
CXCR2	chemokine (C-X-C motif) ligand 2 (Interleukin 8 receptor beta)
DHA	docosahexaenoic acid
DNFB	dinitrofluorobenzene
DRG	dorsal root ganglia
DSCG	disodium cromoglicate
EGCG	epigallocatechin-3-gallate
ENS	enteric nervous system
EPA	eicosapentaenoic acid
ERK	extracellular signal-regulated kinase
ET-1	endothelin 1
FcεRI	high-affinity IgE receptor
FGF	fibroblast growth factor
FODMAPs	fermentable oligosaccharides, disaccharides, monosaccharides and polyols
GATA-1	GATA binding protein-1
GATA-2	GATA binding protein-2
GI	gastrointestinal
GLA	γ-linolenic acid
GM-CSF	granulocyte macrophage colony-stimulating factor
IBD	inflammatory bowel disease
IBS	irritable bowel syndrome
IBS-C	IBS with predominant constipation
IBS-D	IBS with predominant diarrhea
IBS-M	mixed IBS
IBS-U	unclassified IBS
IFN	interferon
Ig	immunoglobulin
IL	interleukin
IP3	inositol-1,4,5-triphosphate
JAM	junctional adhesion molecule
JNK	c-Jun NH2–terminal kinase
LIF	leukemia inhibitory factor
LPS	lipopolysaccharide
LT	leukotriene
MAPK	mitogen-activated protein kinase
MBP	eosinophil major basic protein
MC-CPA	carboxypeptidase A3
MCP	monocyte chemotactic protein
MIP	macrophage inflammatory protein
MMP	matrix metalloproteinase
MMP9	matrix metallopeptidase 9
MRGPRX2	MAS-related G-protein-coupled receptor X2
MS	maternal separation test
MyD88	myeloid differentiation primary response 88
NFκβ	nuclear factor κβ
NGF	nerve growth factor
NK	natural killer
NO	nitric oxide
PAF	platelet activating factor
PAMP	pathogen-associated molecular pattern
PDGF	platelet-derived growth factor
PG	prostaglandin
PI-IBS	post-infectious IBS
PI3K-Akt	phosphoinositide 3-OH kinase-protein kinase B
PKC	protein kinase C
PKC θ	calcium-insensitive protein kinase C theta
PLCγ1	phosphoinositide-specific phospholipase C
pp125 (FAK)	focal adhesion kinase
PPARγ	peroxisome proliferator-activated receptor γ
PUFA	polyunsaturated fatty acid
RANTES	regulated upon activation, normal T cell expressed and secreted
RBL	Rat basophilic leukemia
RBL-2H3	rat basophilic leukemia mast cell line
ROS	reactive oxygen species
S1P1	sphingosine-1-phosphate (S1P) receptor 1
S1P2	sphingosine-1-phosphate (S1P) receptor 2
SCF	stem cell factor
SCFA	short chain fatty acid
SOC	store-operated Ca^2+^ channels
SP	substance P
SphK	sphingosine kinase
SyK	tyrosine-protein kinase SYK or spleen tyrosine kinase
TGF	transforming growth factor
TLR	toll-like receptors
TNF	tumor necrosis factor
Treg	regulatory T cells
VCAM-1	vascular cell adhesion molecule 1
VEGF	vascular endothelial growth factor
VIP	vasoactive intestinal peptide
VDR	vitamin D receptor
WRS	wrap restraint stress

## References

[1] Black C.J., Ford A.C. (2020). Global burden of irritable bowel syndrome: Trends, predictions and risk factors. Nat. Rev. Gastro. Hepat..

[2] Grad S., Dumitrascu D.L. (2020). Irritable Bowel Syndrome Subtypes: New Names for Old Medical Conditions. Dig. Dis..

[3] Creed F. (2019). Review article: The incidence and risk factors for irritable bowel syndrome in population-based studies. Aliment Pharm. Therap..

[4] Canavan C., West J., Card T. (2014). Review article: The economic impact of the irritable bowel syndrome. Aliment Pharm. Therap..

[5] Spiller R., Major G. (2016). IBS and IBD-separate entities or on a spectrum?. Nat. Rev. Gastro. Hepat..

[6] Balmus I.M., Ciobica A., Cojocariu R., Luca A.C., Gorgan L. (2020). Irritable Bowel Syndrome and Neurological Deficiencies: Is There A Relationship? The Possible Relevance of the Oxidative Stress Status. Medicina.

[7] Ng Q.X., Soh A.Y.S., Loke W., Lim D.Y., Yeo W.S. (2018). The role of inflammation in irritable bowel syndrome (IBS). J. Inflamm. Res..

[8] Verne G.N., Price D.D. (2002). Irritable bowel syndrome as a common precipitant of central sensitization. Curr. Rheumatol. Rep..

[9] Casado-Bedmar M., Keita Å.V. (2020). Potential neuro-immune therapeutic targets in irritable bowel syndrome. Therap. Adv. Gastroenter..

[10] Labanski A., Langhorst J., Engler H., Elsenbruch S. (2020). Stress and the brain-gut axis in functional and chronic-inflammatory gastrointestinal diseases: A transdisciplinary challenge. Psychoneuroendocrinology.

[11] Thangam E.B., Jemima E.A., Singh H., Baig M.S., Khan M., Mathias C.B., Church M.K., Saluja R. (2018). The Role of Histamine and Histamine Receptors in Mast Cell-Mediated Allergy and Inflammation: The Hunt for New Therapeutic Targets. Front Immunol..

[12] da Silva E.Z., Jamur M.C., Oliver C. (2014). Mast Cell Function: A New Vision of an Old Cell. Journal of Histochem. Cytochem.

[13] Galli S.J., Borregaard N., Wynn T.A. (2011). Phenotypic and functional plasticity of cells of innate immunity: Macrophages, mast cells and neutrophils. Nat. Immunol..

[14] Gentek R., Ghigo C., Hoeffel G., Bulle M.J., Msallam R., Gautier G., Launay P., Chen J., Ginhoux F., Bajénoff M. (2018). Hemogenic Endothelial Fate Mapping Reveals Dual Developmental Origin of Mast Cells. Immunity.

[15] Li Z., Liu S., Xu J., Zhang X., Han D., Liu J., Xia M., Yi L., Shen Q., Xu S. (2018). Adult Connective Tissue-Resident Mast Cells Originate from Late Erythro-Myeloid Progenitors. Immunity.

[16] Dwyer D.F., Barrett N.A., Austen K.F. (2016). Expression profiling of constitutive mast cells reveals a unique identity within the immune system. Nat. Immunol..

[17] Gurish M.F., Austen K.F. (2012). Developmental Origin and Functional Specialization of Mast Cell Subsets. Immunity.

[18] Collington S.J., Timothy J., Williams T.J., Weller C.L. (2011). Mechanisms underlying the localisation of mast cells in tissues. Trends Immunol..

[19] Galli S.J., Grimbaldeston M., Tsai M. (2008). Immunomodulatory mast cells: Negative, as well as positive, regulators of innate and acquired immunity. Nat. Rev. Immunol..

[20] Iemura A., Tsai M., Ando A., Wershi B.K., Galli S.J. (1994). The c-kit Ligand, Stem Cell Factor, Promotes Mast Cell Survival by Suppressing Apoptosis. Am. J. Pathol..

[21] Hogaboam C., Kunkel S.L., Strieter R.M., Taub D.D., Lincoln P., Standiford T.J., Lukacs N.W. (1998). Novel Role of Transmembrane SCF for Mast Cell Activation and Eotaxin Production in Mast Cell-Fibroblast Interactions. J. Immunol..

[22] Macey M.R., Sturgill J.L., Johanna K., Morales J.K., Falanga Y.T., Morales J., Sarah K., Norton S.K., Yerram N., Shim H. (2010). IL-4 and TGF-b1 Counterbalance One Another while Regulating Mast Cell Homeostasis. J. Immunol..

[23] Weller C.L., Collington S.J., Hartnell A., Conroy D.M., Kaise T., Barker J.E., Wilson M.S., Taylor G.W., Jose P.J., Williams T.J. (2007). Chemotactic action of prostaglandin E2 on mouse mast cells acting via the PGE2 receptor 3. Proc. Nat. Acad. Sci. USA.

[24] Irani A.A., Schechter N.M., Craig S.S., Deblois G., Schwartz L.B. (1986). Two types of human mast cells that have distinct neutral protease compositions. Proc. Nat. Acad. Sci. USA.

[25] Pejler G., Rönnberg E., Waern I., Wernersson S. (2010). Mast cell proteases: Multifaceted regulators of inflammatory disease. Blood.

[26] Kirshenbaum A.S., Swindle E., Kulka M., Wu Y., Metcalfe D.D. (2008). Effect of lipopolysaccharide (LPS) and peptidoglycan (PGN) on human mast cell numbers, cytokine production, and protease composition. BMC Immunol..

[27] Gebhardt T., Lorentz A., Detmer F., Trautwein C., Bektas H., Manns M.P., Bischoff S.C. (2005). Growth, phenotype, and function of human intestinal mast cells are tightly regulated by transforming growth factor β1. Gut.

[28] Kanakura Y., Thompson H., Nakano T., Yamamura T., Asai H., Kitamura Y., Metcalfe D.D., Galli S.J. (1988). Multiple bidirectional alterations of phenotype and changes in proliferative potential during the in vitro and in vivo passage of clonal mast cell populations derived from mouse peritoneal mast cells. Blood.

[29] Galli S.J., Kalesnikoff J., Grimbaldeston M.A., Piliponsky A.M., Williams C., Tsai M. (2005). Mast cells as “tunable” effector and immunoregulatory cells: Recent Advances. Annu. Rev. Immunol..

[30] Subramanian H., Gupta K., Ali H. (2016). Roles of MAS-related G protein coupled receptor-X2 (MRGPRX2) on mast cell-mediated host defense, pseudoallergic drug reactions and chronic inflammatory diseases. Allergy Clin. Immunol..

[31] McNeil B.D., Pundir P., Meeker S., Han L., Undem B.J., Kulka M., Dong X. (2015). Identification of a mast cell specific receptor crucial for pseudo-allergic drug reactions. Nature.

[32] Marshall J.S. (2004). Mast-cell responses to pathogens. Nat. Rev. Immunol..

[33] Plum T., Xi Wang W., Rettel M., Krijgsveld J., Thorsten B., Feyerabend T.B., Rodewald H.R. (2020). Human Mast Cell Proteome Reveals Unique Lineage, Putative Functions, and Structural Basis for Cell Ablation. Immunity.

[34] Metz M., Siebenhaar F., Maurer M. (2008). Mast cell functions in the innate skin immune system. Immunobiology.

[35] Pundir P., Liu R., Vasavda C., Serhan N., Limjunyawong N., Yee R., Zhan Y., Dong X., Wu X., Zhang Y. (2019). A Connective Tissue Mast Cell-Specific Receptor Detects Bacterial Quorum Sensing Molecules and Mediates Antibacterial Immunity. Cell Host Microbe.

[36] Sibilano R., Fross B., Pucillo C.E. (2014). Mast cell activation: A complex interplay of positive and negative signaling pathways. Eur. J. Immunol..

[37] MacGlashan D. (2008). IgE receptor and signal transduction in mast cells and basophils. Cur. Op. Immunol..

[38] Krystel-Whittemore M., Dileepan K.N., Wood J.G. (2016). Mast Cell: A Multi-Functional Master Cell. Front Immunol..

[39] Nakae S., Suto H., Iikura M., Kakurai M., Sedgwick J.D., Tsai M., Galli S.J. (2006). Mast Cells Enhance T Cell Activation: Importance of Mast Cell Costimulatory Molecules and Secreted TNF. J. Immunol..

[40] Wulff B.C., Wilgus T.A. (2013). Mast cell activity in the healing wound: More than meets the eye?. Exp. Dermatol..

[41] Sun J., Sukhova G.K., Yang M., Wolters P.J., MacFarlane L.A., Libby P., Sun C., Zhang Y., Liu J., Ennis T.L. (2007). Mast cells modulate the pathogenesis of elastase-induced abdominal aortic aneurysms in mice. J. Clin. Investig..

[42] Yang M.Q., Ma Y.Y., Tao S.F., Ding J., Rao L.H., Jiang H., Li J.Y. (2014). Mast cell degranulation promotes ischemia reperfusion injury in rat liver. J. Surg. Res..

[43] Kalesnikoff J., Galli S.J. (2008). New developments in mast cell biology. Nat. Immunol..

[44] Gounaris E., Erdman S.E., Restaino C., Gurish M.F., Friend D.S., Gounari F., Lee D.M., Zhang G., Glickman J.N., Shin K. (2007). Mast cells are an essential hematopoietic component for polyp development. Proc. Natl. Acad. Sci. USA.

[45] Uranga J.A., Cámara J.C., Herradón E., Vera G., Jagerovic N., Quesada E., Fernández J., Lombó F., Abalo R., Tyagi A., Prasad S. (2017). New strategies for treatment and prevention of colorectal cancer. Gastrointestinal Cancers.

[46] Sinnamon M.J., Carter K.J., Sims L.P., Lafleur B., Fingleton B., Matrisian L.M. (2008). A protective role of mast cells in intestinal tumorigenesis. Carcinogenesis.

[47] Lovell R.M., Ford A.C. (2012). Global prevalence of and risk factors for irritable bowel syndrome: A meta-analysis. Clin. Gastroenterol. Hepatol..

[48] Schmulson M.J., Drossman D.A. (2017). What Is New in Rome IV. J. Neurogastroenterol..

[49] Fichna J., Fichna J. (2020). A Comprehensive Overview of Irritable Bowel Syndrome-Clinical and Basic Science Aspects.

[50] Buhner S., Schemann M. (2012). Mast cell–nerve axis with a focus on the human gut. BBA-Mol. Basis. Dis..

[51] Costa M., Brookes S.J.H., Hennig G.W. (2000). Anatomy and physiology of the enteric nervous System. Gut.

[52] Yu L.C., Perdue M.H. (2001). Role of mast cells in intestinal mucosal function: Studies in models of hypersensitivity and stress. Immunol. Rev..

[53] Breunig E., Michel K., Florian Zeller F., Stefan Seidl S., Weyhern C.W.H.V., Schemann M. (2007). Histamine excites neurones in the human submucous plexus through activation of H1, H2, H3 and H4 receptors. J. Physiol..

[54] Keely S.J., Stack W.A., O’Donoghue D.P., Baird A.W. (1995). Regulation of ion transport by histamine in human colon. Eur. J. Pharmacol..

[55] Bode H., Schmitz H., Fromm M., Scholz P., Riecken E.O., Schulzke J.D. (1998). IL-1beta and TNF-alpha, but not IFN-alpha, IFN-gamma, IL-6 or IL-8, are secretory mediators in human distal colon. Cytokine.

[56] Schwartz L.B., Lewis R.A., Austen K.F. (1981). Tryptase from Human Pulmonary Mast Cells. J. Biol. Chem..

[57] van der Kleij H.P.M., Bienenstock J. (2005). Significance of conversation between mast cells and nerves. Allergy Asthma Clin. Immunol..

[58] Stead R.H., Dixon M.F., Bramwell N.H., Riddell R.H., Biennenstock J. (1989). Mast cells are closely apposed to nerves in the human gastrointestinal mucosa. Gastroenterology.

[59] Gebhardt T., Gerhard R., Bedoui S., Erpenbeck V.J., Hoffmann M.W., Manns M.P., Bischoff S.C. (2005). β2-Adrenoceptor-mediated suppression of human intestinal mast cell functions is caused by disruption of filamentous actin dynamics. Eur. J. Immunol..

[60] Zhang L., Song J., Hou X. (2016). Mast Cells and Irritable Bowel Syndrome: From the Bench to the Bedside. J. Neurogastroent. Motil..

[61] López Gómez L., Bagués A., Uranga J.A., Abalo R., Fichna J. (2020). Preclinical models of irritable bowel syndrome. A Comprehensive Overview of Irritable Bowel Syndrome-Clinical and Basic Science Aspects.

[62] Vannucchi M.G., Evangelista S. (2018). Experimental Models of Irritable Bowel Syndrome and the Role of the Enteric Neurotransmission. J. Clin. Med..

[63] Overman E.L., Rivier J.E., Moeser A.J. (2012). CRF induces intestinal epithelial barrier injury via the release of mast cell proteases and TNF-α. PLoS ONE..

[64] Taché Y., Larauche M., Yuan P.Q., Million M. (2018). Brain and gut CRF signaling: Biological actions and role in the gastrointestinal tract. Curr. Mol. Pharmacol..

[65] Krammer L., Sowa A.S., Lorentz A. (2019). Mast cells in irritable bowel syndrome: A systematic review. J. Gastroint. Liver. Dis..

[66] Buhner S., Li Q., Vignali S., Barbara G., De Giorgio R., Stanghellini V., Cremon C., Zeller F., Langer R., Daniel H. (2009). Activation of human enteric neurons by supernatants of colonic biopsy specimens from patients with irritable bowel syndrome. Gastroenterology.

[67] Guilarte M., Santos J., de Torres I., Alonso C., Vicario M., Ramos L., Martínez C., Casellas F., Saperas E., Malagelada J.R. (2007). Diarrhoea-predominant IBS patients show mast cell activation and hyperplasia in the jejunum. Gut.

[68] Balestra B., Vicini R., Cremon C., Zecchi L., Dothel G., Vasina V., De Giorgio R., Paccapelo A., Pastoris O., Stanghellini V. (2012). Colonic mucosal mediators from patients with irritable bowel syndrome excite enteric cholinergic motor neurons. Neurogastroent. Motil..

[69] Barbara G., Stanghellini V., De Giorgio R., Cremon C., Cottrell G.S., Santini D., Pasquinelli G., Morselli-Labate A.M., Grady E.F., Bunnett N.W. (2004). Activated mast cells in proximity to colonic nerves correlate with abdominal pain in irritable bowel syndrome. Gastroenterology.

[70] Liang W.J., Zhang G., Luo H.S., Liang L.X., Huang D., Zhang F.C. (2016). Tryptase and Protease-Activated Receptor 2 Expression Levels in Irritable Bowel Syndrome. Gut Liver.

[71] Nasser Y., Boeckxstaens G.E., Wouters M.M., Schemann M., Vanner S. (2014). Using human intestinal biopsies to study the pathogenesis of irritable bowel syndrome. Neurogastroent. Motil..

[72] Camilleri M., Lasch K., Zhou W. (2012). Irritable bowel syndrome: Methods, mechanisms, and pathophysiology. The confluence of increased permeability, inflammation, and pain in irritable bowel syndrome. Am. J. Physiol. Gastrointest. Liver. Physiol..

[73] Park J.H., Rhee P.L., Kim H.S., Lee J.H., Kim Y.H., Kim J.J., Rhee J.C. (2006). Mucosal mast cell counts correlate with visceral hypersensitivity in patients with diarrhea predominant irritable bowel syndrome. J. Gastroen. Hepatol..

[74] O’Sullivan M., Clayton N., Breslin N.P., Harman I., Bountra C., McLaren A., O’Morain C.A. (2000). Increased mast cells in the irritable bowel syndrome. Neurogastroent. Motil..

[75] Cremon C., Carini G., Wang B., Vasina V., Cogliandro R.F., De Giorgio R., Stanghellini V., Grundy D., Tonini M., De Ponti F. (2011). Intestinal serotonin release, sensory neuron activation, and abdominal pain in irritable bowel syndrome. Am. J. Gastroenterol..

[76] Barbara G., Wang B., Stanghellini V., de Giorgio R., Cremon C., Di Nardo G., Trevisani M., Campi B., Geppetti P., Tonini M. (2007). Mast cell-dependent excitation of visceral-nociceptive sensory neurons in irritable bowel syndrome. Gastroenterology.

[77] Valdez-Morales E.E., Overington J., Guerrero-Alba R., Ochoa-Cortes F., Ibeakanma C.O., Spreadbury I., Bunnett N.W., Beyak M., Vanner S.J. (2013). Sensitization of peripheral sensory nerves by mediators from colonic biopsies of diarrhea-predominant irritable bowel syndrome patients: A role for PAR2. Am. J. Gastroenterol..

[78] Cenac N., Andrews C.N., Holzhausen M., Chapman K., Cottrell G., Andrade-Gordon P., Steinhoff M., Barbara G., Beck P., Bunnett N.W. (2007). Role for protease activity in visceral pain in irritable bowel syndrome. J. Clin. Invest..

[79] Klooker T.K., Braak B., Koopman K., Welting O., Wouters M.M., van der Heide S., Schemann M., Bischoff S.C., van den Wijngaard R.N., Boeckxstaens G.E. (2010). The mast cell stabiliser ketotifen decreases visceral hypersensitivity and improves intestinal symptoms in patients with irritable bowel syndrome. Gut.

[80] Stefanini G.F., Prati E., Albini M.C., Piccinini G., Capelli S., Castelli E., Mazzetti M., Gasbarrini G. (1992). Oral disodium cromoglycate treatment on irritable bowel syndrome: An open study on 101 subjects with diarrheic type. Am. J. Gastroenterol..

[81] Stefanini G.F., Saggioro A., Alvisi V., Angelini G., Capurso L., di Lorenzo G., Dobrilla G., Dodero M., Galimberti M., Gasbarrini G. (1995). Oral cromolyn sodium in comparison with elimination diet in the irritable bowel syndrome, diarrheic type. Multicenter study of 428 patients. Scand. J. Gastroenterol..

[82] Ghadir M.R., Poradineh M., Sotodeh M., Ansari R., Kolahdoozan S., Hormati A., Yousefi M.H., Mirzaei S., Vahedi H. (2017). Mesalazine Has No Effect on Mucosal Immune Biomarkers in Patients with Diarrhea-Dominant Irritable Bowel Syndrome Referred to Shariati Hospital: A Randomized Double-Blind, Placebo-Controlled Trial. Middle East J. Dig. Dis..

[83] Camilleri M. (2013). Current and future pharmacological treatments for diarrhea-predominant irritable bowel syndrome. Expert Opin. Pharmaco..

[84] Zhang F.M., Li S., Ding L., Xiang S.H., Zhu H.T., Yu J.H., Xu G.Q. (2019). Effectiveness of mesalazine to treat irritable bowel syndrome: A meta-analysis. Medicine.

[85] Cheng W., Li J., Liu X. (2020). 5-Aminosalicylic acid for treatment of irritable bowel syndrome: A protocol for a systematic review and meta-analysis. Medicine.

[86] Tack J.F., Jr Miner P.B., Fischer L., Harris M.S. (2011). Randomised clinical trial: The safety and efficacy of AST-120 in non-constipating irritable bowel syndrome—a double-blind, placebo-controlled study. Aliment Pharmacol. Ther..

[87] Wouters M.M., Balemans D., Van Wanrooy S., Dooley J., Cibert-Goton V., Alpizar Y.A., Valdez-Morales E.E., Nasser Y., Van Veldhoven P.P., Vanbrabant W. (2016). Histamine receptor H1-mediated sensitization of TRPV1 mediates visceral hypersensitivity and symptoms in patients with irritable bowel syndrome. Gastroenterology.

[88] Fabisiak A., Włodarczyk J., Fabisiak N., Storr M., Fichna J. (2017). Targeting Histamine Receptors in Irritable Bowel Syndrome: A Critical Appraisal. J. Neurogastroent. Motil..

[89] Vivinus-Nébot M., Dainese R., Anty R., Saint-Paul M.C., Nano J.L., Gonthie N.R., Marjoux S., Frin-Mathy G., Bernard G., Hébuterne X. (2012). Combination of allergic factors can worsen diarrheic irritable bowel syndrome: Role of barrier defects and mast cells. Am. J. Gastroenterol..

[90] Piche T., Barbara G., Aubert P., des Varannes S.B., Dainese R., Nano J.L., Cremon C., Stanghellini V., de Giorgio R., Galmiche J.P. (2009). Impaired intestinal barrier integrity in the colon of patients with irritable bowel syndrome: Involvement of soluble mediators. Gut.

[91] Gecse K., Roka R., Ferrier L., Leveque M., Eutamene H., Cartier C., Ait-Belgnaoui A., Rosztoczy A., Izbeki F., Fioramonti J. (2008). Increased faecal serine protease activity in diarrhoeic IBS patients: A colonic lumenal factor impairing colonic permeability and sensitivity. Gut.

[92] Barbaro M.R., Fuschi D., Cremon C., Carapelle M., Dino P., Marcellini M.M., Dothel G., de Ponti F., Stanghellini V., Barbara G. (2018). Escherichia coli Nissle 1917 restores epithelial permeability alterations induced by irritable bowel syndrome mediators. Neurogastroent. Motil..

[93] Nébot-Vivinus M., Harkat C., Bzioueche H., Cartier C., Plichon-Dainese R., Moussa L., Eutamene H., Pishvaie D., Holowacz S., Seyrig C. (2014). Multispecies probiotic protects gut barrier function in experimental models. World J. Gastroenterol..

[94] Annaházi A., Ferrier L., Bézirard V., Levêque M., Eutamène H., AitBelgnaoui A., Coëffier M., Ducrotté P., Roka R., Inczefi O. (2013). Luminal cysteine-proteases degrade colonic tight junction structure and are responsible for abdominal pain in constipation-predominant IBS. Am. J. Gastroenterol..

[95] Wilcz-Villega E.M., McClean S., O’Sullivan M.A. (2013). Mast cell tryptase reduces junctional adhesion molecule-A (JAM-A) expression in intestinal epithelial cells: Implications for the mechanisms of barrier dysfunction in irritable bowel syndrome. Am. J. Gastroenterol..

[96] Jacob C., Yang P.C., Darmoul D., Amadesi S., Saito T., Cottrell G.S., Coelho A.M., Singh P., Grady E.F., Perdue M. (2005). Mast cell tryptase controls paracellular permeability of the intestine. Role of protease-activated receptor 2 and beta-arrestins. J. Biol. Chem..

[97] Spiller R., Campbell E. (2006). Post-infectious irritable bowel syndrome. Curr. Opin. Gastroenterol..

[98] Gwee K.A., Collins S.M., Read N.W., Rajnakova A., Deng Y., Graham J.C., McKendrick M.W., Moochhala S.M. (2003). Increased rectal mucosal expression of interleukin 1β in recently acquired post-infectious irritable bowel syndrome. Gut.

[99] Uno Y. (2019). Hypothesis: Mechanism of irritable bowel syndrome in inflammatory bowel disease. Med. Hypotheses.

[100] Rej A., Sanders D.S. (2018). Gluten-Free Diet and Its ‘Cousins’ in Irritable Bowel Syndrome. Nutrients.

[101] Halmos E.P., Power V.A., Shepherd S.J., Gibson P.R., Muir J.G. (2014). A diet low in FODMAPs reduces symptoms of irritable bowel syndrome. Gastroenterolog..

[102] Mansueto P., Seidita A., D’Alcamo A., Carroccio A. (2015). Role of FODMAPs in Patients With Irritable Bowel Syndrome. Nutr. Clin. Pract..

[103] Altobelli E., Del Negro V., Angeletti P.M., Latella G. (2017). Low-FODMAP Diet Improves Irritable Bowel Syndrome Symptoms: A Meta-Analysis. Nutrient..

[104] McIntosh K., Reed D.E., Schneider T., Dang F., Keshteli A.H., De Palma G., Madsen K., Bercik P., Vanner S. (2017). FODMAPs alter symptoms and the metabolome of patients with IBS: A randomised controlled trial. Gut..

[105] Whelan K., Martin L.D., Staudacher H.M., Lomer M.C.E. (2018). The low FODMAP diet in the management of irritable bowel syndrome: An evidence-based review of FODMAP restriction, reintroduction and personalisation in clinical practice. J. Hum. Nutr. Diet..

[106] Kamphuis J.B., Guiard B., Leveque M., Olier M., Jouanin I., Yvon S., Tondereau V., Rivière P., Guéraud F., Chevolleau S. (2020). Lactose and fructo-oligosaccharides increase visceral sensitivity in mice via glycation processes, increasing mast cell density in colonic mucosa. Gastroenterology.

[107] Chen B.R., Du L.J., He H.Q., Kim J.J., Zhao Y., Zhang Y.W., Luo L., Dai N. (2017). Fructo-oligosaccharide intensifies visceral hypersensitivity and intestinal inflammation in a stress-induced irritable bowel syndrome mouse model. World J. Gastroenterol..

[108] Rej A., Sanders D.S. (2019). The overlap of irritable bowel syndrome and noncoeliac gluten sensitivity. Curr. Opin. Gastroenterol..

[109] Frossi B., De Carli M., Calabrò A. (2019). Coeliac Disease and Mast Cells. Int. J. Mol. Sci..

[110] Tuck C.J., Vanner S.J. (2017). Dietary therapies for functional bowel symptoms: Recent advances, challenges, and future directions. Neurogastroent. Motil..

[111] Bischoff S.C. (2007). Role of mast cells in allergic and non-allergic immune responses: Comparison of human and murine data. Nat. Rev. Immunol..

[112] Siebenhaar F., Redegeld F.A., Bischoff S.C., Gibbs B.F., Maurer M. (2018). Mast cells as drivers of disease and therapeutic targets. Trends Immunol..

[113] Yu Y., Blokhuis B.R., Garssen J., Redegeld F.A. (2016). Non-IgE mediated mast cell activation. Eur. J. Pharmacol..

[114] Simren M., Månsson A., Langkilde A.M., Svedlund J., Abrahamsson H., Bengtsson U., Björnsson E.S. (2001). Food-related gastrointestinal symptoms in the irritable bowel syndrome. Digestion.

[115] Choung R.S., Talley N.J. (2006). Food allergy and intolerance in IBS. Gastroen Hepatol..

[116] Virta L.J., Ashorn M., Kolho K.L. (2013). Cow’s milk allergy, asthma, and pediatric IBD. J. Pediatr. Gastroen. Nutr..

[117] Walker M.M., Powell N., Talley N.J. (2014). Atopy and the gastrointestinal tract—a review of a common association in unexplained gastrointestinal disease. Expert Rev. Gastroen. Hepatol..

[118] Mansueto P., D’Alcamo A., Seidita A., Carroccio A. (2015). Food allergy in irritable bowel syndrome: The case of non-celiac wheat sensitivity. World J. Gastroenterol..

[119] Bashashati M., Moossavi S., Cremon C., Barbaro M.R., Moraveji S., Talmon G., Rezaei N., Hughes P.A., Bian Z.X., Choi C.H. (2018). Colonic immune cells in irritable bowel syndrome: A systematic review and meta-analysis. Neurogastroent. Motil..

[120] Robles A., Perez Ingles D., Myneedu K., Deoker A., Sarosiek I., Zuckerman M.J., Schmulson M.J., Bashashati M. (2019). Mast cells are increased in the small intestinal mucosa of patients with irritable bowel syndrome: A systematic review and meta-analysis. Neurogastroent. Motil..

[121] Boeckxstaens G. (2015). Mast cells and inflammatory bowel disease. Curr. Opin. Pharmacol..

[122] De Zuani M., Dal Secco C., Frossi B. (2018). Mast cells at the crossroads of microbiota and IBD. Eur. J. Immunol..

[123] de Haan J.J., Hadfoune M., Lubbers T., Hodin C., Lenaerts K., Ito A., Verbaeys I., Skynner M.J., Cailotto C., van der Vliet J. (2013). Lipid-rich enteral nutrition regulates mucosal mast cell activation via the vagal anti-inflammatory reflex. Am. J. Physiol. Gastrointest. Liver. Physiol..

[124] Hagemann P.M., Nsiah-Dosu S., Hundt J.E., Hartmann K., Orinska Z. (2019). Modulation of mast cell reactivity by lipids: The neglected side of allergic diseases. Front Immunol..

[125] Schumann J., Basiouni S., Gück T., Fuhrmann H. (2014). Treating canine atopic dermatitis with unsaturated fatty acids: The role of mast cells and potential mechanisms of action. J. Anim. Physiol. Anim. Nutr..

[126] van den Elsen L.W., Nusse Y., Balvers M., Redegeld F.A., Knol E.F., Garssen J., Willemsen L.E. (2013). n-3 Long-chain PUFA reduce allergy-related mediator release by human mast cells in vitro via inhibition of reactive oxygen species. Br. J. Nutr..

[127] Park B., Park S., Park J., Park M., Min T., Jin M. (2013). Omega-3 fatty acids suppress Th2-associated cytokine gene expressions and GATA transcription factors in mast cells. J. Nut. Biochem..

[128] Obata T., Nagakura T., Masaki T., Maekawa K., Yamashita K. (1999). Eicosapentaenoic acid inhibits prostaglandin D2 generation by inhibiting cyclo-oxygenase-2 in cultured human mast cells. Clin. Exp. Allergy.

[129] van Diest S.A., van den Elsen L.W., Klok A.J., Welting O., Hilbers F.W., van de Heijning B.J., Gaemers I.C., Boeckxstaens G.E., Werner M.F., Willemsen L.E. (2015). Dietary marine n-3 PUFAs do not affect stress-induced visceral hypersensitivity in a rat maternal separation model. J. Nutr..

[130] Gueck T., Seidel A., Fuhrmann H. (2003). Effects of essential fatty acids on mediators of mast cells in culture. Prostag. Leukotr. Ess..

[131] Gueck T., Seidel A., Baumann D., Meister A., Fuhrmann H. (2004). Alterations of mast cell mediator production and release by gamma-linolenic and docosahexaenoic acid. Vet. Dermatol..

[132] Gueck T., Seidel A., Fuhrmann H. (2004). Consequences of eicosapentaenoic acid (n-3) and arachidonic acid (n-6) supplementation on mast cell mediators. J. Anim. Physiol. Anim. Nutr..

[133] Ju H.R., Wu H.Y., Nishizono S., Sakono M., Ikeda I., Sugano M., Imaizumi K. (1996). Effects of dietary fats and curcumin on IgE-mediated degranulation of intestinal mast cells in brown Norway rats. Biosci. Biotechnol. Biochem..

[134] Vinolo M.A., Rodrigues H.G., Nachbar R.T., Curi R. (2011). Regulation of inflammation by short chain fatty acids. Nutrients.

[135] Leonel A.J., Alvarez-Leite J.I. (2012). Butyrate: Implications for intestinal function. Curr. Opin. Clin. Nutr. Metab. Care..

[136] Wang C.C., Wu H., Lin F.H., Gong R., Xie F., Peng Y., Feng J., Hu C.H. (2018). Sodium butyrate enhances intestinal integrity, inhibits mast cell activation, inflammatory mediator production and JNK signaling pathway in weaned pigs. Innate. Immun..

[137] Martínez V., Iriondo De-Hond A., Borrelli F., Capasso R., Del Castillo M.D., Abalo R. (2020). Cannabidiol and Other Non-Psychoactive Cannabinoids for Prevention and Treatment of Gastrointestinal Disorders: Useful Nutraceuticals?. Int. J. Mol. Sci..

[138] De Filippis D., Esposito G., Cirillo C., Cipriano M., de Winter B., Scuderi C., Sarnelli G., Cuomo R., Steardo L., de Man J. (2011). Cannabidiol reduces intestinal inflammation through the control of neuroimmune axis. PLoS ONE.

[139] De Filippis D., Negro L., Vaia M., Cinelli M.P., Iuvone T. (2013). New insights in mast cell modulation by palmitoylethanolamide. CNS Neurol. Disord. Drug Targets.

[140] Cerrato S., Brazis P., della Valle M.F., Miolo A., Puigdemont A. (2010). Effects of palmitoylethanolamide on immunologically induced histamine, PGD2 and TNFalpha release from canine skin mast cells. Vet. Immunol. Immunopathol..

[141] Cantarella G., Scollo M., Lempereur L., Saccani-Jotti G., Basile F., Bernardini R. (2011). Endocannabinoids inhibit release of nerve growth factor by inflammation-activated mast cells. Biochem. Pharmacol..

[142] Mazzari S., Canella R., Petrelli L., Marcolongo G., Leon A. (1996). N-(2-hydroxyethyl) hexadecanamide is orally active in reducing edema formation and inflammatory hyperalgesia by down-modulating mast cell activation. Eur. J. Pharmacol..

[143] De Filippis D., D’Amico A., Cinelli M.P., Esposito G., Di Marzo V., Iuvone T. (2009). Adelmidrol, a palmitoylethanolamide analogue, reduces chronic inflammation in carrageenin granuloma model in rat. J. Cell Mol. Med..

[144] De Filippis D., D’Amico A., Cipriano M., Petrosino S., Orlando P., Di Marzo V., Iuvone T. (2010). Levels of endocannabinoids and palmitoylethanolamide and their pharmacological manipulation in chronic granulomatous inflammation in rats. Pharmacol. Res..

[145] De Filippis D., Luongo L., Cipriano M., Palazzo E., Cinelli M.P., de Novellis V., Maione S., Iuvone T. (2011). Palmitoylethanolamide reduces granuloma-induced hyperalgesia by modulation of mast cell activation in rats. Mol. Pain..

[146] Costa B., Comelli F., Bettoni I., Colleoni M.P., Giagnoni G. (2008). The endogenous fatty acid amide, palmitoylethanolamide, has anti-allodynic and anti-hyperalgesic effects in a murine model of neuropathic pain: Involvement of CB1, TRPV1 and PPARgamma receptors and neurotrophic factors. Pain.

[147] Esposito E., Paterniti I., Mazzon E., Genovese T., Di Paola R., Galuppo M., Cuzzocrea S. (2011). Effects of palmitoylethanolamide on release of mast cell peptidases and neurotrophic factors after spinal cord injury. Brain. Behav. Immun..

[148] Scarampella F., Abramo F., Noli C. (2001). Clinical and histological evaluation of an analogue of palmitoylethanolamide, PLR 120 (comicronized Palmidrol INN) in cats with eosinophilic granuloma and eosinophilic plaque: A pilot study. Vet. Dermatol..

[149] Cremon C., Stanghellini V., Barbaro M., Cogliandro R., Bellacosa L., Santos J., Vicario M., Pigrau M., Alonso Cotoner C., Lobo B. (2017). Randomised clinical trial: The analgesic properties of dietary supplementation with palmitoylethanolamide and polydatin in irritable bowel syndrome. Alim. Pharmacol. Ther..

[150] Olivera A., Rivera J. (2005). Sphingolipids and the balancing of immune cell function: Lessons from the mast cell. J. Immunol..

[151] Chiba N., Masuda A., Yoshikai Y., Matsuguchi T. (2007). Ceramide inhibits LPS-induced production of IL-5, IL-10, and IL-13 from mast cells. J. Cell. Physiol..

[152] Jolly P.S., Bektas M., Olivera A., Gonzalez-Espinosa C., Proia R.L., Rivera J., Milstien S., Spiegel S. (2004). Transactivation of sphingosine-1-phosphate receptors by FcepsilonRI triggering is required for normal mast cell degranulation and chemotaxis. J. Exp. Med..

[153] Liu Z.Q., Li X.X., Qiu S.Q., Yu Y., Li M.G., Yang L.T., Li L.J., Wang S., Zheng P.Y., Liu Z.G. (2017). Vitamin D contributes to mast cell stabilization. Allergy.

[154] Zingg J. (2007). Vitamin E and mast cells. Vitam. Horm..

[155] Gueck T., Aschenbach J.R., Fuhrmann H. (2002). Influence of vitamin E on mast cell mediator release. Vet. Dermatol..

[156] Ranadive N.S., Lewis R. (1982). Differential effects of antioxidants and indomethacin on compound 48/80 induced histamine release and Ca2+ uptake in rat mast cells. Immunol. Lett..

[157] Lecleire S., Hassan A., Marion-Letellier R., Antonietti M., Savoye G., Bole-Feysot C., Lerebours E., Ducrotte P., Dechelotte P., Coeffier M. (2008). Combined glutamine and arginine decrease proinflammatory cytokine production by biopsies from Crohn’s patients in association with changes in nuclear factor-kappa B and p38 mito-gen-activated protein kinase pathways. J. Nutr..

[158] Lechowski S., Feilhauer K., Staib L., Coeffier M., Bischoff S.C., Lorentz A. (2013). Combined arginine and glutamine decrease release of de novo synthesized leukotrienes and expression of proinflammatory cytokines in activated human intestinal mast cells. Eur. J. Nutr..

[159] Zhu H., Pi D., Leng W., Wang X., Hu C.A., Hou Y., Xiong J., Wang C., Qin Q., Liu Y. (2017). Asparagine preserves intestinal barrier function from LPS-induced injury and regulates CRF/CRFR signaling pathway. Innate. Immun..

[160] van Bergenhenegouwen J., Braber S., Loonstra R., Buurman N., Rutten L., Knipping K., Savelkoul P., Harthoorn L., Jahnsen F., Garssen J. (2018). Oral exposure to the free amino acid glycine inhibits the acute allergic response in a model of cow’s milk allergy in mice. Nut. Res..

[161] Sakai S., Sugawara T., Matsubara K., Hirata T. (2009). Inhibitory effect of carotenoids on the degranulation of mast cells via suppression of antigen-induced aggregation of high affinity IgE receptors. J. Biol. Chem..

[162] Kim H., Ahn Y., Lee G., Cho S., Kim J., Lee C., Lim B., Ju S., An W. (2015). Effects of astaxanthin on dinitrofluorobenzene-induced contact dermatitis in mice. Mol. Med. Rep..

[163] Sato Y., Akiyama H., Suganuma H., Watanabe T., Nagaoka M.H., Inakuma T., Goda Y., Maitani T. (2004). The feeding of -carotene down-regulates serum IgE levels and inhibits the type I allergic response in mice. Biol. Pharm. Bull..

[164] Kinoshita T., Koike K., Mwamtemi H.H., Ito S., Ishida S., Nakazawa Y., Kurokawa Y., Sakashita K., Higuchi T., Takeuchi K. (2000). Retinoic acid is a negative regulator for the differentiation of cord blood-derived human mast cell progenitors. Blood.

[165] Hjertson M., Kivinen P., Dimberg L., Nilsson K., Harvima I., Nilsson G. (2003). Retinoic acid inhibits in vitro development of mast cells but has no marked effect on mature human skin tryptase- and chymase-positive mast cells. J. Investig. Dermatol..

[166] Ishida S., Kinoshita T., Sugawara N., Yamashita T., Koike K. (2003). Serum inhibitors for human mast cell growth: Possible role of retinol. Eur. J. Allergy. Clin. Immunol..

[167] Astorquiza M.I., Helle B., Vergara R.E. (1980). Effect of vitamin A onthe in vitro degranulation of mouse mastcells. Allergol. Immunopathol..

[168] Middleton E., Drzewiecki G. (1984). Flavonoid inhibition of human basophil histamine release stimulated by various agents. Biochem. Pharmacol..

[169] Trnovsky J., Letourneau R., Haggag E., Boucher W., Theoharides T.C. (1993). Quercetin-induced expression of rat mast cell protease II and accumulation of secretory granules in rat basophilic leukemia cells. Biochem. Pharmacol..

[170] Alexandrakis M., Singh L., Boucher W., Letourneau R., Theofilopoulos P., Theoharides T.C. (1999). Differential effect of flavonoids on inhibition of secretion and accumulation of secretory granules in rat basophilic leukemia cells. Int. J. Immunopharmacol..

[171] Kimata M., Inagaki N., Nagai H. (2000). Effects of luteolin and other flavonoids on IgE-mediated allergic reactions. Planta. Med..

[172] Kimata M., Shichijo S., Miura T., Serizawa I., Inagaki N., Nagai H. (2000). Effects of luteolin, quercetin and baicalein on immunoglobulin E-mediated mediator release from human cultured mast cells. Clin. Exp. Allergy.

[173] Seelinger G., Merfort I., Schempp C.M. (2008). Anti-oxidant, anti-inflammatory and anti-allergic activities of luteolin. Planta. Med..

[174] Park H.H., Lee S., Son K.Y., Park S.B., Kim M.S., Choi E.J., Singh T.S., Ha J.H., Lee M.G., Kim J.E. (2008). Flavonoids inhibit histamine release and expression of proinflammatory cytokines in mast cells. Arch. Pharm. Res..

[175] Kempuraj D., Madhappan B., Chrístodoulou S., Boucher W., Cao J., Papadopoulou N., Cetrulo C.L., Theoharides T.C. (2005). Flavonols inhibit proinflammatory mediator release, intracellular calcium ion levels and protein kinase C phosphorylation in human mast cells. Br. J. Pharmacol..

[176] Yang Y., Oh J.M., Heo P., Shin J.Y., Kong B., Shin J., Lee J.C., Oh J.S., Park K.W., Lee C.H. (2013). Polyphenols differentially inhibit degranulation of distinct subsets of vesicles in mast cells by specific interaction with granule-type-dependent SNARE complexes. Biochem. J..

[177] Hagenlocher Y., Feilhauer K., Schäffer M., Bischoff S.C., Lorentz A. (2017). Citrus peel polymethoxyflavones nobiletin and tangeretin suppress LPS- and IgE-mediated activation of human intestinal mast cells. Eur. J. Nutr..

[178] Tanaka T., Iuchi A., Harada H., Hashimoto S. (2019). Potential Beneficial Effects of Wine Flavonoids on Allergic Diseases. Diseases.

[179] Hagenlocher Y., Gommeringer S., Held A., Feilhauer K., Köninger J., Bischoff S.C., Lorentz A. (2019). Nobiletin acts anti-inflammatory on murine IL-10-/- colitis and human intestinal fibroblasts. Eur. J. Nutr..

[180] Hubert J., Berger M., Nepveu F., Paul F., Daydé J. (2008). Effects of fermentation on the phytochemical composition and antioxidant properties of soy germ. Food Chem..

[181] Moussa L., Bézirard V., Salvador-Cartier C., Bacquié V., Houdeau E., Théodorou V. (2013). A new soy germ fermented ingredient displays estrogenic and protease inhibitor activities able to prevent irritable bowel syndrome-like symptoms in stressed female rats. Clin. Nutr..

[182] Inoue T., Suzuki Y., Ra C. (2010). Epigallocatechin-3-gallate inhibits mast cell degranulation, leukotriene C4 secretion, and calcium influx via mitochondrial calcium dysfunction. Free Radic. Biol. Med..

[183] Inoue T., Suzuki Y., Ra C. (2011). Epigallocatechin-3-gallate induces cytokine production in mast cells by stimulating an extracellular superoxide-mediated calcium influx. Biochem. Pharmacol..

[184] Khan N., Mukhtar H. (2018). Tea Polyphenols in promotion of human health. Nutrients.

[185] Matsuo N., Yamada K., Shoji K., Mori M., Sugano M. (1997). Effect of tea polyphenols on histamine release from rat basophilic leukemia (RBL-2H3) cells: The structure-inhibitory activity relationship. Allergy.

[186] Yamashita K., Suzuki Y., Matsui T., Yoshimaru T., Yamaki M., Suzuki-Karasaki M., Hayakawa S., Shimizu K. (2000). Epigallocatechin gallate inhibits histamine release from rat basophilic leukemia (RBL-2H3) cells: Role of tyrosine phosphorylation. Biochem. Biophys. Res. Commun..

[187] Murata K., Takano S., Masuda M., Iinuma M., Matsuda H. (2013). Anti-degranulating activity in rat basophilic leukemia RBL-2H3 cells of flavanone glycosides and their aglycones in citrus fruits. J. Nat. Med..

[188] Fiorani M., Accorsi A., Blasa M., Diamantini G., Piatti E. (2006). Flavonoids from Italian multi-floral honeys reduce the extracellular ferricyanide in human red blood cells. J. Agric. Food Chem..

[189] Guendouz M., Haddi A., Grar H., Kheroua O., Saidi D., Kaddouri H. (2017). Preventive effects of royal jelly against anaphylactic response in a murine model of cow’s milk allergy. Pharm. Biol..

[190] Gupta S.C., Kismali G., Aggarwal B.B. (2013). Curcumin, a component of turmeric: From farm to pharmacy. Biofactors.

[191] Lee J.H., Kim J.W., Ko N.Y., Mun S.H., Her E., Kim B.K., Han J.W., Lee H.Y., Beaven M.A., Kim Y.M. (2008). Curcumin, a constituent of curry, suppresses IgE-mediated allergic response and mast cell activation at the level of Syk. J. Allergy Clin. Immunol..

[192] Hanai H., Iida T., Takeuchi K., Watanabe F., Maruyama Y., Andoh A., Tsujikawa T., Fujiyama Y., Mitsuyama K., Sata M. (2006). Curcumin maintenance therapy for ulcerative colitis: Randomized, multicenter, double-blind, placebo-controlled trial. Clin. Gastroen. Hepatol..

[193] Lang A., Salomon N., Wu J.C., Kopylov U., Lahat A., Har-Noy O., Ching J.Y., Cheong P.K., Avidan B., Gamus D. (2015). Curcumin in combination with mesalamine induces remission in patients with mild-to-moderate ulcerative colitis in a randomized controlled trial. Clin. Gastroen. Hepatol..

[194] Bundy R., Walker A.F., Middleton R.W., Booth J. (2004). Turmeric extract may improve irritable bowel syndrome symptomology in otherwise healthy adults: A pilot study. J. Altern. Complement. Med..

[195] Portincasa P., Bonfrate L., Scribano M.L., Kohn A., Caporaso N., Festi D., Campanale M.C., Di Rienzo T., Guarino M., Taddia M. (2016). Curcumin and Fennel Essential Oil Improve Symptoms and Quality of Life in Patients with Irritable Bowel Syndrome. J. Gastrointestin. Liver. Dis..

[196] Hagenlocher Y., Bergheim I., Zacheja S., Schäffer M., Bischoff S.C., Lorentz A. (2013). Cinnamon extract inhibits degranulation and de novo synthesis of inflammatory mediators in mast cells. Allergy.

[197] Hagenlocher Y., Hösel A., Bischoff S., Lorentz A. (2016). Cinnamon extract reduces symptoms, inflammatory mediators and mast cell markers in murine IL-10−/− colitis. J. Nut. Biochem..

[198] Hagenlocher Y., Kiessling K., Schäffer M., Bischoff S.C., Lorentz A. (2015). Cinnamaldehyde is the main mediator of cinnamon extract in mast cell inhibition. Eur. J. Nutr..

